# Endothelial Klf9 fine-tunes Akt signaling to act as a transcriptional brake restraining retinal angiogenesis

**DOI:** 10.7150/ijbs.133293

**Published:** 2026-05-11

**Authors:** Honglian Wu, Tianjing Yang, Yuning Xun, Ming Zhou, Yutian Zhang, Xueming Yao, Yongsheng Chang, Yi Lei, Hua Yan

**Affiliations:** 1 School of Medicine, Nankai University, Tianjin, 300071, China.; 2 Department of Ophthalmology, Tianjin Medical University General Hospital, Ministry of Education International Joint Laboratory of Ocular Diseases, Tianjin Key Laboratory of Ocular Trauma, Tianjin Institute of Eye Health and Eye Diseases, Laboratory of Molecular Ophthalmology, Tianjin Medical University, Tianjin 300070, China.; 3 Department of Physiology and Pathophysiology, School of Basic Medical Sciences, Key Laboratory of Immune Microenvironment and Disease (Ministry of Education), Tianjin Key Laboratory of Cellular Homeostasis and Disease, Tianjin Medical University, Tianjin 300070, China.

**Keywords:** Klf9, Endothelial cells, Angiogenesis, Akt signaling

## Abstract

Retinal angiogenesis requires precise transcriptional regulation. Krüppel-like factor 9 (Klf9) has been implicated in various biological processes; however, its specific role in retinal vascular development and ocular neovascular disease remains unclear. In this study, we identified Klf9 as a critical transcriptional regulator of retinal vascular homeostasis. Spatiotemporal transcriptomic and single-cell RNA sequencing analyses revealed that Klf9 was highly enriched in retinal endothelial cells and upregulated during vascular maturation. Using genetic mouse models, we demonstrated that endothelial-specific Klf9 deletion accelerated neonatal retinal vascular expansion and tip cell formation, whereas its overexpression delayed angiogenesis and disrupted barrier function. In oxygen-induced retinopathy, Klf9 loss exacerbated pathological neovascularization and leakage, while its overexpression conferred protection. Integrated RNA-seq and ATAC-seq profiling of human retinal microvascular endothelial cells revealed that Klf9 represses a network of genes involved in the PI3K-Akt pathway and focal adhesions. Key effectors, including *AKT1*, *PTK2*, and *RAC1,* were suppressed by reduced chromatin accessibility at their promoters. Both *in vitro* and *in vivo* rescue experiments confirmed that Akt activation reverses vascular hypoplasia caused by Klf9 overexpression, whereas Akt inhibition normalizes the hyper-angiogenic phenotype of the Klf9-deficient endothelium. Collectively, these findings establish Klf9 as a transcriptional brake on retinal angiogenesis, acting through chromatin-mediated suppression of the PI3K-Akt pathway, and provide new mechanistic insights and potential therapeutic targets for pathological retinal angiogenesis.

## Introduction

Angiogenesis is a tightly controlled, stage-dependent process that is essential for tissue development and homeostasis 1. Endothelial cells (ECs) initially adopt a highly migratory and proliferative state to drive vascular sprouting but subsequently transition toward quiescence, stabilization, and pruning to generate a functional vascular network 2. While extensive work has defined the signaling pathways that promote endothelial activation—most notably VEGF (vascular endothelial growth factor), Notch, and PI3K-Akt—far less is known about the transcriptional and epigenetic mechanisms that actively restrain angiogenic growth and enforce vascular maturation 3.

This is particularly critical in the retina, a metabolically demanding neural tissue that requires precise vascular patterning and strict barrier integrity. During postnatal development, retinal angiogenesis proceeds through stereotypical phases of expansion, remodeling, and pruning 4. Failure to terminate endothelial activation properly results in excessive and unstable vessels that underlie vision-threatening diseases such as proliferative diabetic retinopathy (PDR) and retinopathy of prematurity (ROP) 5. Although anti-VEGF therapies are clinically effective, their incomplete efficacy and limited effect on vascular stabilization suggest that additional VEGF-independent regulatory mechanisms remain to be defined 5.

Transcription factors and chromatin regulators are increasingly recognized as central determinants of endothelial state transitions, as they convert transient extracellular cues into durable gene-expression programs 6. Among these, the Krüppel-like factor (KLF) family functions as a systems-level regulatory hub integrating mechanical, metabolic, and inflammatory signals to govern endothelial activation, quiescence, and barrier function 7-9. Several KLFs, including Klf2, Klf4, and Klf15, are implicated in vascular biology. For instance, Klf2 is potently induced by laminar shear stress and confers an atheroprotective and anti-inflammatory phenotype to ECs 10. Klf4 interacts with the Notch pathway to modulate tip and stalk cell selection during sprouting angiogenesis 11. Klf15 negatively regulates retinal angiogenesis by activating Notch1 signaling 12. However, whether additional KLF family members act as transcriptional brakes to terminate angiogenic growth during vascular maturation remains unclear.

Krüppel-like factor 9 (Klf9) is a compelling candidate for this function. Klf9 is highly expressed in the retina and has been shown in other tissues to restrain proliferation, regulate metabolic adaptation, and enforce cellular differentiation through transcriptional repression and chromatin modulation 13-15. In neural, cardiac, and cancer contexts, Klf9 functions as a context-dependent growth suppressor, suggesting that it may similarly constrain endothelial activation 13, 15-23. However, its roles in vascular development and pathological angiogenesis remain unclear.

Pathological angiogenesis is characterized by the sustained activation of proangiogenic signaling pathways, among which the PI3K-Akt axis serves as a central driver of endothelial proliferation, migration, cytoskeletal remodeling, and survival 24-27. Although PI3K-Akt signaling is classically regulated at the level of receptor activation and kinase phosphorylation, whether this pathway is coordinately constrained at the transcriptional and chromatin levels during vascular maturation is poorly understood 24.

In this study, we identified Klf9 as a stage-specific transcriptional brake of retinal angiogenesis. By integrating endothelial-specific genetic models, human disease data, single-cell and bulk transcriptomics, chromatin accessibility profiling, and *in vivo* and *in vitro* functional rescue experiments, we demonstrated that Klf9 was selectively upregulated during retinal vascular maturation and suppressed during pathological neovascularization. Mechanistically, Klf9 directly repressed chromatin accessibility in key PI3K-Akt and focal adhesion genes, thereby limiting endothelial activation. Loss of Klf9 accelerates developmental angiogenesis and exacerbates pathological neovascularization, whereas its overexpression confers vascular protection. Together, these findings establish a chromatin-encoded Klf9-PI3K-Akt regulatory axis that couples angiogenic growth with vascular stabilization and identify Klf9 as a potential therapeutic target for ischemic retinopathies.

## Materials and Methods

### Mice

All experimental procedures involving vertebrates were performed in strict accordance with the National Institutes of Health (NIH) Guide for the Care and Use of Laboratory Animals (8th Edition). The study protocol was reviewed and approved by the Institutional Animal Care and Use Committee of Tianjin Medical University. Wild-type and transgenic mice were maintained in a C57BL/6J genetic background. Mice were housed in a specific pathogen-free facility under rigorously controlled environmental conditions: a 12-h light/dark cycle, an ambient temperature of 22 ± 1°C, and a relative humidity of 50 ± 10%. Animals received a standard autoclaved rodent chow diet and filtered water ad libitum. Homozygous *Klf9^fl/fl^* mice were crossed with heterozygous *Cdh5-cre* transgenic mice to generate endothelial-specific Klf9 knockout mice (*Klf9^∆EC^*), which express Cre recombinase under the control of the vascular endothelial-cadherin promoter 13, 28. Specifically, *CDH5-cre* heterozygous (*CDH5-cre+/-*) mice were paired with *Klf9 flox/flox* homozygous mice to generate F1 offspring with the genotype *CDH5-cre+/-; Klf9 flox/+* (heterozygous). The F1 mice were then crossed with *Klf9 flox/flox* homozygous mice to obtain the target experimental group (*CDH5-cre+/-; Klf9 flox/flox*, designated as *Klf9^ΔEC^* mice). Littermates with the genotype *CDH5-cre-/-; Klf9 flox/flox* were used as the control group (designated as *Klf9^fl/fl^* mice). Endothelial-specific Klf9 overexpression mice (*Klf9^iEC-Tg^*) were generated by crossing *Rosa26-Klf9^flox/flox^* mice with *Cdh5-(PAC)-CreERT2* transgenic mice 13, 29. *Rosa26-Klf9^flox/flox^* mice were generated using the CRISPR-Cas9 system to insert the CAG-LoxP-STOP-LoxP-Klf9 cassette into the mouse Rosa26 locus 13. *Cdh5-(PAC)-CreERT2* heterozygous (*CDH5-creERT2+/-*) mice were crossed with *Rosa26-Klf9^flox/flox^* homozygous mice to generate the experimental group (*CDH5-creERT2+/-; Rosa26-Klf9 flox/+*, designated as *Klf9^iEC-Tg^* mice). Littermates with the genotype *CDH5-creERT2-/-; Rosa26-Klf9 flox/+* (referred to as* Klf9^R-loxp^* mice) were used as controls.

For *Klf9^∆EC^* and *Klf9^fl/fl^* mice, pups were sacrificed at postnatal day 7 (P7) for early assessment of retinal vessel growth, and at P12 and P21 for evaluation of retinal vessel maturation. In the oxygen-induced retinopathy (OIR) mouse model, *Klf9^∆EC^* and *Klf9^fl/fl^* mice were sacrificed at P17 and P19 to assess retinal angiogenesis progression. For *Klf9^iEC-Tg^* mice, neonatal pups received intraperitoneal (i.p.) injections of tamoxifen (60 µg per neonate) once daily from P1 to P3. Control mice (*Klf9^R-loxp^*) received identical tamoxifen injections to control potential off-target effects of the drug. In the OIR mouse model, *Klf9^iEC-Tg^* and *Klf9^R-loxp^* mice received i.p. injections of tamoxifen (100 µg per neonate) once daily from P10 to P12 and were sacrificed at P17 and P19. Real-time PCR was performed to identify the genotypes of transgenic mice. To functionally rescue the observed phenotypes, the AKT inhibitor MK2206 (10 mg/kg, Beyotime, S1078) or AKT activator SC79 (20 mg/kg, Beyotime, S7863) was administered via intraperitoneal injection daily from postnatal days 4 to 6 (P4-P6). *Klf9^∆EC^* and *Klf9^fl/fl^* littermates received MK2206, while *Klf9^iEC-Tg^* and *Klf9^R-loxp^* littermates received SC79. Pups were sacrificed at P7 for subsequent retinal analyses. Anesthesia was induced by isoflurane inhalation, and euthanasia was performed by cervical dislocation.

### Magnetic bead-based isolation of mouse retinal ECs

Freshly dissected retinas were collected in pre-cooled MACS (Magnetic Activated Cell Sorting) buffer, minced, and digested with 1 mg/mL collagenase IV (containing 100 U/mL DNase I) at 37°C for 10-15 min with intermittent pipetting. Digestion was stopped with 10% fetal bovine serum (FBS) medium, and the suspension was filtered through a 40-μm strainer and centrifuged at 300 g for 5 min. Cells were resuspended in MACS buffer, counted, and adjusted to ~1×10⁸ cells/mL. The cell suspension was incubated with CD45 microbeads at 4°C for 15 min. After washing, the cells were applied to an LS column under the OctoMACS magnetic field. The flow-through containing CD45⁻ cells was collected, and the column was washed three times with buffer to maximize recovery of unlabeled cells. The pooled CD45⁻ cell fraction was then centrifuged at 300 g for 5 min and resuspended in MACS buffer. For endothelial cell isolation, CD45⁻ cells were incubated with CD31 microbeads at 4°C for 15 min. After washing, the cells were applied to a new LS column under a magnetic field. The column was washed three times, and CD31⁺ cells were eluted with buffer after removal from the magnet. Purified endothelial cells were pelleted by centrifugation, processed for RNA extraction, or stored until further use.

### Validation of endothelial-specific Klf9 knockout and overexpression in mice

CD31⁺ endothelial cells were isolated from the retinas of Klf9^ΔEC^ mice, Klf9^iEC-Tg^ mice, and their respective controls at P14 using magnetic bead-based separation. Total RNA was extracted, reverse transcribed, and subjected to RT-qPCR to determine the relative expression levels of Klf9, thereby validating the efficiency of endothelial-specific knockout or overexpression.

### OIR model

The OIR model was established as previously described 30. At P7, the entire mouse litter and nursing dams were exposed to 75% oxygen in a Plexiglas chamber with an oxygen controller (Pro-Ox 110, Biospherix). The dams were rotated daily to minimize oxygen toxicity. At P12, the pups were returned to the room air. At P17, the pups were euthanized, and retinas were harvested for whole-mount immunostaining, as described below. Quantification included the central avascular area and area occupied by pathological neovascular tufts, each expressed as a percentage of the total retinal area.

### Retinal whole-mount immunostaining

The mice were sacrificed at specific postnatal days (P7, P12, and P21 for neonatal mice and P17 and P19 for OIR mice) by cervical dislocation. The eyes were enucleated and fixed in 4% paraformaldehyde (PFA) for 20-30 min at room temperature (RT), and the retinas were dissected into four leaflets. The retinas were washed thrice for 15 min each with PBS. They were then permeabilized and blocked simultaneously by incubating in PBS containing 1% FBS, 3% bovine serum albumin (BSA, Fraction V), and 0.5% Triton X-100 for 2 h at RT on a shaker. Retinas were incubated with primary antibodies diluted in the blocking solution for 48-72 h at 4°C on a shaker. The following antibodies were used: ERG (Abcam, ab92513, 1:200), Ki67 (Cell Signaling Technology, 9129S, 1:200), Col IV (Abcam, ab6586, 1:200), NG2 (Abcam, ab275024, 1:200) and Ter119 (BioLegend, 116203, 1:100). After primary incubation, retinas were washed twice for 1 h each with PBS containing 0.1% Triton X-100 (PBS-T) at RT to remove unbound antibodies. Subsequently, the retinas were incubated with appropriate secondary antibodies (donkey anti-rabbit IgG Alexa Fluor 555, donkey anti-rat IgG Alexa Fluor 647, Invitrogen, 1:500) and Isolectin B4 (IB4, Thermo Fisher Scientific, I21411 / I21413, 1:100) diluted in blocking solution for 2 h at RT in the dark. Following incubation with the secondary antibody, the retinas were washed again (twice for 1 h each with PBS-T). Four radial cuts were made to flatten the retina, which were then mounted with the ganglion cell layer up on a glass slide using Vectashield antifade mounting medium (Vector Laboratories, H-1000). High-resolution z-stack images covering the entire retina or specific quadrants were acquired using a Zeiss LSM 800 confocal microscope, and maximum intensity projections of the z-stacks were generated. The laser power, gain, and offset were maintained constant across the samples. Vascular parameters were quantified manually or using ImageJ software (NIH) with the AngloTool plugin. The avascular area was defined as the central region devoid of IB4-positive capillaries and was expressed as a percentage of the total retinal area. Neovascular tufts were defined as abnormal focal clusters of ECs projecting into the vitreous, and their areas were quantified.

### Cell culture and treatments

Human retinal microvascular endothelial cells (HRMECs) were cultured in an Endothelial Cell Medium (ECM, ScienCell, USA), supplemented with 5% FBS. The cells were maintained at 37°C in a humidified incubator with 5% CO₂. For hypoxia experiments, HRMECs at 80-90% confluence were washed with PBS. The culture medium was replaced with fresh, pre-warmed, or pre-equilibrated ECM. The cells were then placed in a hypoxic incubator (1% O₂, 5% CO₂) for 24 h. Normoxic control cells were maintained in a standard incubator (21% O₂, 5% CO₂) for the same duration. The cells from both groups were harvested for western blot analysis.

### Adenoviral transduction

Klf9 knockdown was achieved using a validated adenoviral vector expressing short hairpin RNA (shRNA) that targets human Klf9 (Ad-shKlf9). Klf9 overexpression was performed using an adenovirus carrying the full-length human Klf9 cDNA (Ad-Klf9). Scrambled shRNA (Ad-shCtrl) or an empty adenoviral vector (Ad-Ctrl) served as controls. HRMECs at 70% confluence were transduced with adenoviruses at a multiplicity of infection of 50 in serum-reduced ECM (2% FBS). After 4-6 h, the viral medium was replaced with the complete ECM medium. For rescue experiments, 24 h after viral infection, the medium was replaced with fresh complete ECM containing the AKT inhibitor MK2206 (5 µg/mL, Beyotime, S1078) or the AKT activator SC79 (5 µg/mL, Beyotime, S7863). The cells were stimulated for 24 h.

### EdU proliferation assay

HRMECs were seeded onto glass coverslips in 24-well plates at a density of 2 × 10⁴ cells/well. After respective treatments, cells were pulsed with 10 µM 5-ethynyl-2′-deoxyuridine (EdU) for 2 h at 37°C. The cells were then fixed with 4% PFA for 15 min, permeabilized with 0.5% Triton X-100 for 20 min, and stained using the Click-iT Plus EdU Alexa Fluor 594 Imaging Kit (Thermo Fisher Scientific, C10639) according to the manufacturer's protocol. Nuclei were counterstained with Hoechst 33342 (5 µg/mL) for 10 min. Coverslips were mounted on slides using the ProLong Gold Antifade Mountant. Five random, non-overlapping fields per well were captured using a 20× objective on a fluorescence microscope. The percentage of EdU-positive cells (red nuclei) relative to the total number of Hoechst-positive nuclei (blue) was automatically quantified using ImageJ software.

### Scratch wound healing assay

HRMECs were seeded in 6-well plates and grown to 100% confluence. A sterile 200-µL pipette tip was used to create a uniform, linear scratch through the monolayer. The wells were gently washed with PBS to remove the detached cells, and fresh ECM was added. The wound areas were photographed immediately (0 h) and at 18 h post-scratch using an inverted phase-contrast microscope (Nikon Eclipse Ts2) with a 4× objective. The migration rate was quantified by measuring the remaining cell-free area at each time point using the “Wound Healing Size Tool” plugin in ImageJ. The percentage of wound closure was calculated as: ([Area at T0 - Area at Tx] / Area at T0) × 100%.

### RNA extraction and quantitative PCR

Total RNA was extracted using the TRIzol reagent (Sigma, #101254514) from snap-frozen mouse retinas homogenized with a pellet pestle or from cultured HRMEC monolayers, following the manufacturer's single-phase separation protocol. Briefly, 1 mL of TRIzol was used per 50-100 mg of tissue or per 10⁶ cells. Chloroform (0.2 mL per 1 mL TRIzol) was added, samples were vortexed vigorously, and phases were separated by centrifugation at 12,000 × g for 15 min at 4°C. The aqueous RNA-containing phase was transferred to a new tube, and RNA was precipitated with an equal volume of isopropanol. The RNA pellet was washed twice with 75% ethanol, air dried, and dissolved in RNase-free water. RNA concentration and purity (A260/A280 ratio >1.9, A260/A230 ratio >2.0) were determined using a NanoDrop One spectrophotometer. One microgram of total RNA was reverse-transcribed into cDNA using a High-Capacity cDNA Reverse Transcription Kit (Applied Biosystems, 4368814). Quantitative PCR (qPCR) was performed in triplicate 10-µL reactions using SYBR Green PCR Master Mix (Bio-Rad, 1725274) on a QuantStudio 3 Real-Time PCR System. The relative mRNA expression was calculated using the comparative 2^(-ΔΔCt)^ method, with normalization to the housekeeping genes *Gapdh* (mouse) or *ACTB* (human). The primer sequences used are listed in Supplementary Table 1.

### Western blot

Retinal tissues or HRMEC pellets were lysed in ice-cold RIPA lysis buffer (MilliporeSigma, 20-188) supplemented with 1× Halt Protease and Phosphatase Inhibitor Cocktail (Thermo Fisher Scientific, 78440). The tissues were homogenized using a motorized homogenizer, and the cells were lysed by incubation on ice for 30 min with intermittent vortexing. Lysates were centrifuged at 14,000 × g for 15 min at 4°C to remove insoluble debris. The supernatant was collected, and the protein concentration was determined using the BCA Protein Assay Kit (Pierce, 23225) with BSA as a standard. Equal amounts of protein (20-30 µg) were mixed with 4× Laemmli sample buffer containing 2-mercaptoethanol, heated at 95°C for 15 min, and then separated by SDS-polyacrylamide gel electrophoresis on 10% or 12% Mini-PROTEAN TGX Precast Gels (Bio-Rad) at 120 V for approximately 90 min. Proteins were electrophoretically transferred onto a 0.45-µm PVDF (Polyvinylidene Difluoride) membrane (MilliporeSigma, IPVH304F0) using a wet transfer system (Bio-Rad) at 300 mA for 120 min. The membranes were blocked with 5% non-fat dry milk in Tris-buffered saline containing 0.1% Tween-20 (TBST) for 2 h at RT. The membranes were then incubated overnight at 4°C with primary antibodies diluted in 5% BSA in TBST: rabbit anti-Klf9 (Thermo Fisher, 701888, 1:1000), mouse anti-ACTB (Proteintech, 66009-1-Ig, 1:5000), and mouse anti-GAPDH (Proteintech, 60004-1-Ig, 1:5000). After three 10-min washes with TBST, the membranes were incubated with horseradish peroxidase (HRP)-conjugated secondary antibodies (goat anti-rabbit IgG-HRP, sc-2004, or goat anti-mouse IgG-HRP, sc-2005, Santa Cruz Biotechnology, 1:5000) diluted in 5% milk in TBST for 2 h at RT. After another three washes, the protein bands were visualized using enhanced chemiluminescence Prime Western Blotting Detection Reagent (Cytiva, RPN2232) and imaged using the ChemiDoc MP Imaging System (Bio-Rad). Band intensities were quantified using ImageJ software (NIH), and Klf9 protein expression was normalized to the GAPDH loading control.

### RNA-sequencing and bioinformatics

Total RNA was extracted from HRMECs transduced with Ad-shCtrl, Ad-shKlf9, Ad-Ctrl, and Ad-Klf9 (n=3 independent biological replicates per group) using the RNeasy Mini Kit (Qiagen, 74104), including an on-column DNase I digestion step to remove genomic DNA. RNA integrity was verified using an Agilent 2100 Bioanalyzer; all samples had RNA integrity numbers greater than 8.0. Sequencing libraries were prepared from 1 µg of high-quality total RNA using the Illumina TruSeq Stranded mRNA Library Prep Kit. The poly (A)-containing mRNA was purified using oligo (dT) magnetic beads, followed by fragmentation and first-strand cDNA synthesis using random hexamers. Second-strand cDNA synthesis incorporates dUTP to preserve strand specificity. The cDNA fragments were end-repaired, adenylated, and ligated using unique dual-index adapters. The adapter-ligated fragments were PCR-amplified (15 cycles), and the final libraries were purified and validated for size distribution using an Agilent TapeStation. Libraries were quantified by qPCR (KAPA Library Quantification Kit) and pooled at equimolar ratios. The pool was sequenced on an Illumina NovaSeq 6000 platform (SP flow cell) to generate 150 bp paired-end reads, aiming for a depth of 40-50 million reads per sample.

### Bioinformatic analysis for RNA-Seq

Raw reads were assessed using FastQC v0.11.9 and filtered using Trimmomatic v0.39 (LEADING:20, TRAILING:20, SLIDINGWINDOW:4:20, MINLEN:36). MultiQC v1.11 generated consolidated reports. Clean reads were aligned to GRCh38 using HISAT2 v2.2.1 (--dta, --rna-strandness RF). Alignments were processed with SAMtools v1.12, and gene-level counts were obtained using featureCounts v2.0.1 (-t exon -g gene_id -s 2 -p). DESeq2 v1.34.0, in R v4.1.2, was used for the differential expression analysis. Genes with an adjusted p-value (padj) < 0.05 were considered significant. Differentially expressed genes were identified for Klf9 knockdown vs. control and overexpression vs. control. Venn diagrams identified genes with opposite expression trends between the two comparisons. KEGG (Kyoto Encyclopedia of Genes and Genomes) enrichment of inversely regulated genes was performed using clusterProfiler v4.2.2 (pvalueCutoff = 0.05, qvalueCutoff = 0.05). Chord diagrams (using the circlize R package) and clustering heatmaps (using the pheatmap R package) were generated to visualize gene-pathway associations and expression patterns of key pathways, including focal adhesion and PI3K-Akt.

### Assay for transposase-accessible chromatin with sequencing (ATAC-Seq)

ATAC-seq was performed on freshly harvested HRMECs (50,000 viable cells per sample, n=3 per group) as previously described 31, with minor modifications. Cells were washed with cold PBS and lysed in cold lysis buffer (10 mM Tris-HCl, pH 7.4, 10 mM NaCl, 3 mM MgCl_2_, and 0.1% Igepal CA-630) to isolate the nuclei. The nuclei were immediately tagmented using the Nextera Tn5 Transposase (Illumina, 20034197) for 30 min at 37°C. The tagmented DNA was purified using a MinElute PCR Purification Kit (Qiagen, 28004). The transposed DNA fragments were amplified by limited-cycle PCR (determined by qPCR) using the NEBNext High-Fidelity 2x PCR Master Mix and custom Nextera PCR primers. The final libraries were purified using AMPure XP beads (Beckman Coulter), quantified, and assessed for quality using an Agilent Bioanalyzer (expected smear between 100-1000 bp). Libraries were sequenced on an Illumina NovaSeq 6000 (150 bp paired-end, ~50 million reads/sample).

### Data analysis for ATAC-Seq

Raw reads were assessed with FastQC v0.11.9 and filtered using Trimmomatic v0.39 (parameters as in RNA-seq). Clean reads were aligned to GRCh38 using Bowtie2 v2.4.2 (--very-sensitive -X 2000). Alignments were processed with SAMtools v1.12. PCR duplicates were removed using Picard v2.25.0. Reads with MAPQ < 30 were filtered, and only properly paired reads were retained. Mitochondrial reads and blacklisted regions were masked using Bedtools v2.30.0. Peaks were called using MACS2 v2.2.7.1 (-f BAMPE -g hs -q 0.05 --shift -100 --extsize 200 --nomodel --call-summits). Differential accessibility analysis was performed using DiffBind v3.2.7 (false discovery rate < 0.05). Peak annotation was performed using ChIPseeker v1.28.3. Metagene profiles around the transcription start sites (TSS) and the chromosomal distribution of accessibility changes were generated. KEGG enrichment of genes associated with decreased accessibility after Klf9 overexpression was performed using clusterProfiler v4.2.2 (parameters as in RNA-seq). Chord diagrams were generated to visualize gene-pathway associations. Genes showing concordant changes at both the chromatin accessibility (ATAC-seq) and transcription (RNA-seq) levels were identified as candidate targets for validation.

### Chromatin immunoprecipitation followed by qPCR

HRMECs (~10⁷ cells per immunoprecipitation) were cross-linked with 1% formaldehyde for 10 min at RT. The reaction was quenched by adding glycine to a final concentration of 0.125 M. The cells were washed with cold PBS, scraped, and pelleted. Chromatin was sheared to an average size of 200-500 bp using a Covaris S220 sonicator. The sheared chromatin was pre-cleared using Protein G magnetic beads (Pierce) for 1 h. An aliquot (10 µL) was saved as the "Input" control. The remaining chromatin was incubated overnight at 4°C with 2 µg of anti-Klf9 antibody (Abcam, ab227920) or an equivalent amount of normal rabbit IgG (Cell Signaling Technology, 2729S) as a negative control. Antibody-chromatin complexes were captured by incubation with Protein G magnetic beads for 2 h at 4°C. Beads were washed sequentially with low-salt, high-salt, LiCl, and TE buffers. Cross-links were reversed by incubation with 200 mM NaCl at 65°C overnight. DNA was purified using a QIAquick PCR Purification Kit (Qiagen, Hilden, Germany). The enrichment of specific genomic regions was quantified by qPCR using SYBR Green and primers designed to flank the predicted Klf9 binding motifs in the promoters of the target genes. Results were calculated as a percentage of input using the following formula: % Input = 2^(Ct[Input] - Ct[IP])^ × dilution factor × 100 and were normalized to the IgG control.

### Statistical analysis

All quantitative data are presented as mean ± Standard Error of the Mean from at least three independent biological replicates (n ≥ 3, n represents biological replicates), as indicated in the Fig. legends. Statistical analyses were performed using GraphPad Prism software (version 10.0.0). Normality of the data distribution was assessed using the Shapiro-Wilk test. For comparisons between the two groups, an unpaired two-tailed Student's t-test was used. For comparisons among three or more groups, one-way analysis of variance was performed, followed by Tukey's multiple comparison test. All experiments were successfully replicated on at least two separate occasions.

## Results

### Klf9 expression is upregulated during retinal vascular development in mice

**Figure 1:**
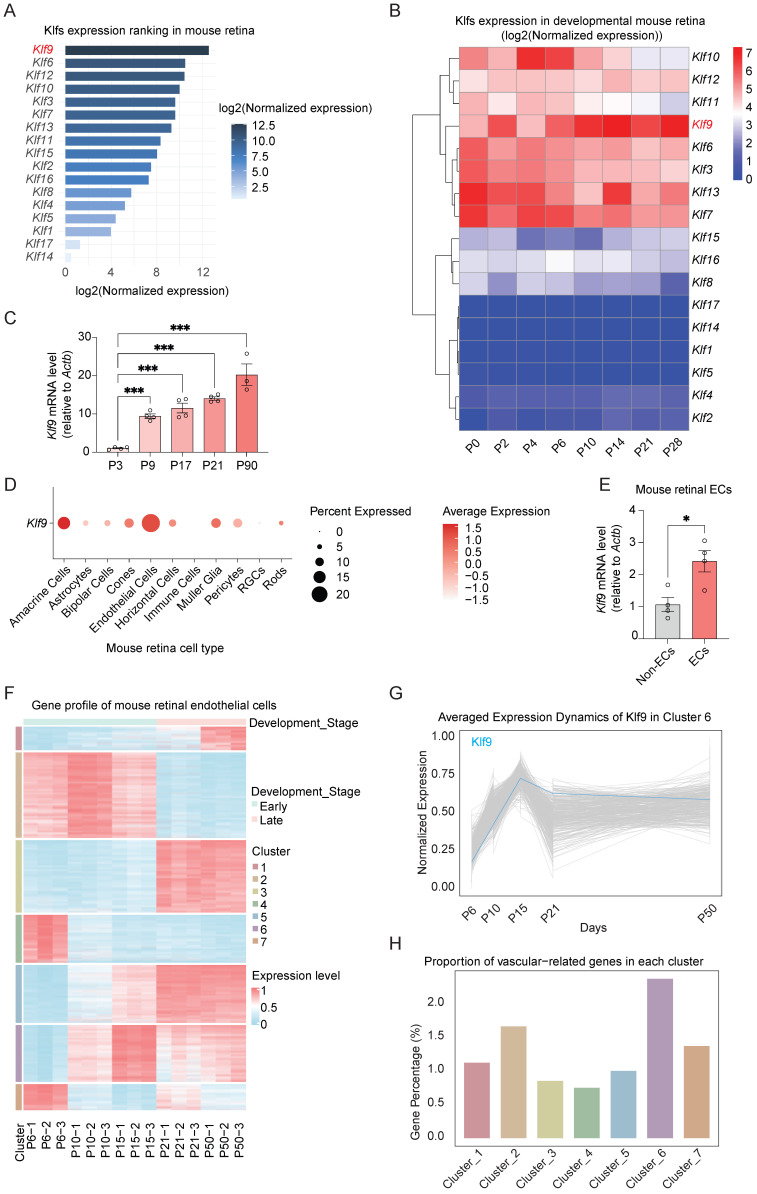
** Klf9 increased during mouse retinal vessel development.** (A) Bar chart depicting KLF family expression (data from GSE234447) in the mouse retina. (B) Heatmap showing the expression of KLF family in the developing mouse retina (data from GSE101986). (C) Relative *Klf9* mRNA expression levels at P3, P9, P17, P21, and P90 (n = 4, 4, 4, 4, and 3, respectively) in the developing mouse retina analyzed by RT-qPCR. ***p < 0.001, mean ± SEM, one-way ANOVA with Tukey's multiple comparisons test. (D) Dot plot showing the percentage of cells expressing Klf9 and the average expression levels across different retinal cell types (data from GSE150703). (E) Relative Klf9 mRNA expression levels between endothelial cells (ECs, n = 4) and non-endothelial cells (non-ECs, n = 4) in the mouse retina analyzed by RT-qPCR. *p < 0.05, mean ± SEM, two-tailed unpaired Student's t test. (F) Heatmap showing gene expression profiles of mouse retinal ECs at various developmental stages, with clusters representing gene co-expression patterns analyzed by k-means method (data from GSE86788). (G) Line graph illustrating the average expression dynamics of Klf9 in Cluster 6 across various developmental time points (data from GSE86788). (H) Bar plot representing the proportion of vascular-related genes in each cluster of retinal ECs (data from GSE86788).

First, we examined the expression of the KLF family members in the developing mouse retina using publicly available data (GSE234447) and found that among the KLF family members, Klf9 exhibited the highest expression level (Fig. 1A) 32. To better investigate the characteristics of the KLF family in retinal development, we analyzed KLF gene expression profiles across various developmental stages within the retina. Heatmap analysis of KLF gene expression in the developing mouse retina (data from GSE101986) revealed distinct temporal patterns 33. Notably, Klf9 showed a sharp increase in expression during the early postnatal stages (Postnatal day 0-14, P0-P14), followed by stabilization at a high level during later developmental stages (P21-P28) (Fig. 1B). RT-qPCR was then used to validate the temporal expression patterns of Klf9 across different retinal developmental stages. Klf9 expression increased approximately tenfold from P3 to P9 (Fig. 1C).

Furthermore, cell-type-specific expression analysis of single-cell RNA sequencing data (GSE150703) 34 revealed that Klf9 was predominantly expressed in ECs, with the highest expression levels observed in ECs compared with other retinal cell types (Fig. 1D). We sorted mouse ECs from the retina to further confirm that* Klf9* expression was significantly enriched in ECs relative to non-ECs (Supplementary Fig. 1A; Fig. 1E). During mouse retinal vascular development (P0 to P21), angiogenesis-related genes undergo significant transcriptional fluctuations. To investigate whether endothelial cell-derived Klf9 might be involved in vascular development, we analyzed a transcriptomic dataset of mouse retinal ECs across different developmental stages (data from GSE86788) 6. In this dataset, the endothelial genes were clustered into seven groups based on their expression patterns (Fig. 1F). Klf9 was assigned to Cluster 6, and its expression increased rapidly from P6 to P15 and was then stabilized at P21, with comparable expression levels observed at P21 and P50 (Fig. 1G). This expression pattern was consistent with the progressive functional maturation of retinal vessels during the second postnatal week, suggesting that Klf9 may play a role in regulating retinal vascular maturation. Cluster 6 contained the highest number of genes associated with vascular function (Fig. 1H). Taken together, Klf9 exhibited high and temporally regulated expression in the developing mouse retina and was predominantly enriched in ECs. Its expression pattern closely parallels that of the vascular-associated genes, suggesting a critical regulatory role in retinal vascular development.

### Endothelial-specific deletion of Klf9 strengthens neonatal retinal vascular development

**Figure 2:**
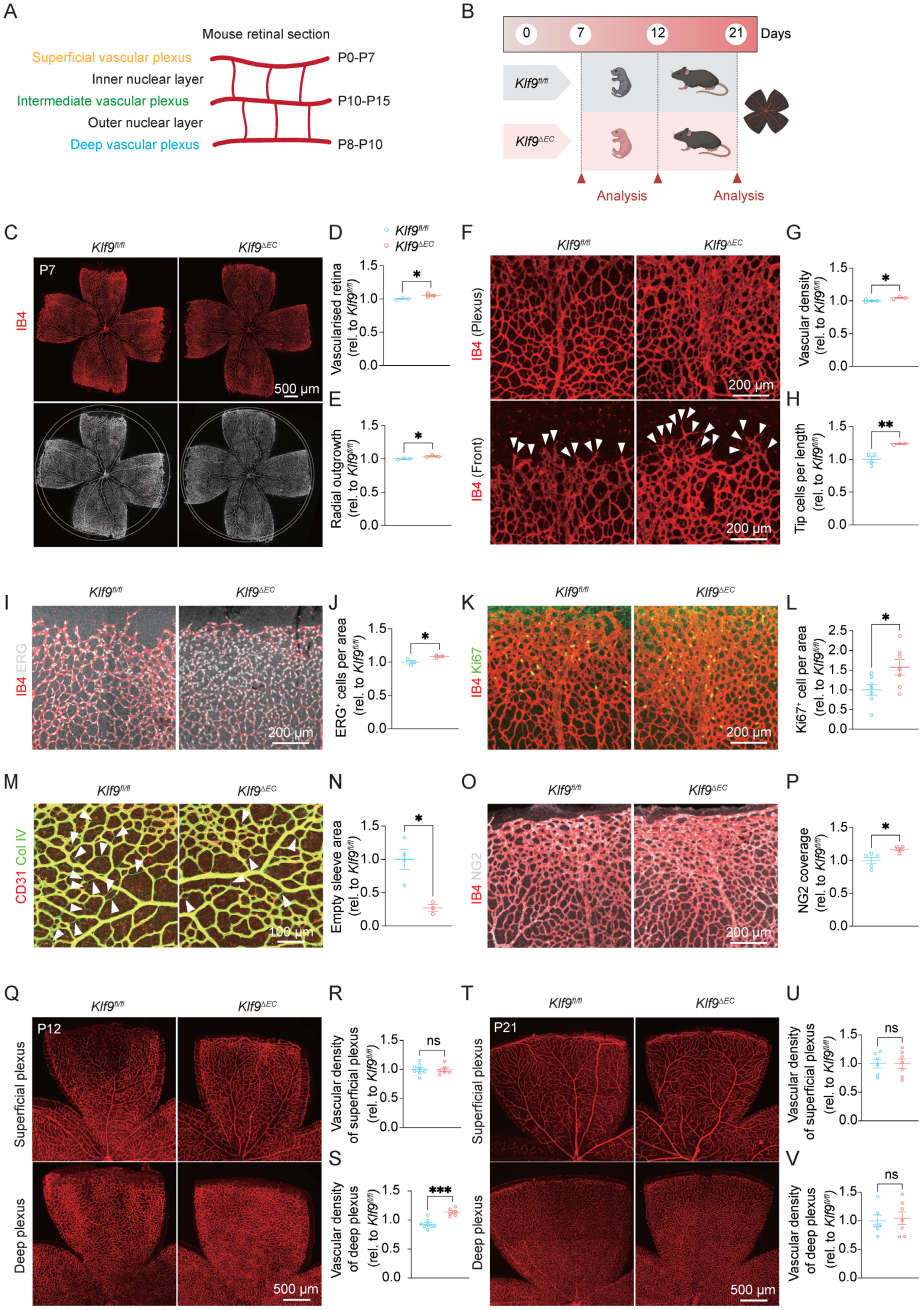
** Klf9 deficiency in ECs promotes neonatal retinal vascular development*.*** (A) Schematic diagram showing the timeline of mouse retinal vascular plexus development, including the superficial, intermediate, and deep layers. (B) Experimental design illustrating the time points for retinal collection and analysis of *Klf9^fl/fl^* and *Klf9^ΔEC^* mice at P7, P12, and P21. (C) Representative images of retinal flat mounts from *Klf9^fl/fl^* and *Klf9^ΔEC^* mice at P7, stained with Isolectin B4 (IB4, red) to visualize the vasculature. (D, E) Quantification of the retinal vascularized area and radial outgrowth in *Klf9^fl/fl^* (n = 3) and *Klf9^ΔEC^* (n = 3) mice at P7. *p < 0.05, mean ± SEM, two-tailed unpaired Student's t test. (F) High-magnification images of the retinal vascular front showing vascular density and tip cells (arrowheads) in the same retinas of *Klf9^fl/fl^* and *Klf9^ΔEC^* retinas. (G, H) Quantification of vascular density and tip cell numbers in *Klf9^fl/fl^* (n = 4) and *Klf9^ΔEC^* (n = 3) mice at P7. *p < 0.05, **p < 0.01, mean ± SEM, two-tailed unpaired Student's t test. (I, J) Representative immunostaining and quantification of ERG expression (gray) in endothelial nuclei of *Klf9^fl/fl^* (n = 3) and *Klf9^ΔEC^* (n = 3) retinas at P7. *p < 0.05, mean ± SEM, two-tailed unpaired Student's t test. (K, L) Co-immunostaining of retinal vasculature (IB4, red) with proliferating cells (Ki67, green) at P7 and quantification of Ki67-positive ECs in *Klf9^fl/fl^* (n = 7) and *Klf9^ΔEC^* (n = 7) retinas. *p < 0.05, mean ± SEM, two-tailed unpaired Student's t test. (M, N) Co-immunostaining of retinal vasculature (IB4, red) with collagen IV (green) indicating vascular regression (arrowheads) in *Klf9^fl/fl^* (n = 4) and *Klf9^ΔEC^* (n = 3) retinas; quantification shown in (N). *p < 0.05, mean ± SEM, two-tailed unpaired Student's t test. (O, P) Co-immunostaining of retinal vasculature (IB4, red) with NG2 (grey) indicating pericyte coverage in *Klf9^fl/fl^* (n = 5) and *Klf9^ΔEC^* (n = 4) retinas; quantification shown in (P). *p < 0.05, mean ± SEM, two-tailed unpaired Student's t test. (Q-S) Representative images of superficial and deep vascular plexuses at P12 and corresponding quantifications of vascular density in *Klf9^fl/fl^* (n = 8) and *Klf9^ΔEC^* (n = 6) retinas. ***p < 0.001, mean ± SEM, two-tailed unpaired Student's t test. (T-V) Representative images and quantification of superficial and deep plexuses at P21 in *Klf9^fl/fl^* (n = 6) and *Klf9^ΔEC^* (n = 7) mice. Mean ± SEM, two-tailed unpaired Student's t test.

The murine retinal vasculature develops in a stereotypical sequence. At birth, ECs migrate from the optic nerve to the retinal surface and expand radially to establish the superficial vascular plexus. After P7, sprouting vessels invade the outer plexus layer to form the deep plexus; between P11 and P12, vessels from the deep plexus ascend into the inner plexus layer to generate the intermediate plexus, with complete vascularization typically achieved by approximately P15 (Fig. 2A) 35. To investigate the role of Klf9 in ECs during neonatal retinal vascular development, we examined the retinal vasculature in *Klf9^fl/fl^* and *Klf9^ΔEC^* mice at various postnatal days (P7, P12, and P21) (Fig. 2B). RT-qPCR was used to determine the knockout efficiency of Klf9 in mouse retinal ECs (Supplementary Fig. 1B).

At P7, *Klf9^ΔEC^* mice exhibited a significantly larger vascularized area compared with *Klf9^fl/fl^* mice. Retinal flat mount images stained with IB4 demonstrated a more extensive vasculature in *Klf9^ΔEC^* mice, with a higher percentage of the total retinal area occupied by vessels and stronger radial outgrowth at P7 (Fig. 2C-E). We further analyzed the retinal vascular front at P7 for vascular density and tip cell formation. High-magnification images revealed increased vascular density and a higher number of tip cells in *Klf9^ΔEC^* retinas (Fig. 2F-H). Additionally, ERG immunostaining of endothelial-cell nuclei revealed a significant increase in EC number in *Klf9^ΔEC^* mice compared with *Klf9^fl/fl^* controls (Fig. 2I, J). Immunostaining for the proliferation marker Ki67 showed significantly higher proliferation of ECs in *Klf9^ΔEC^* retinas at P7 (Fig. 2K, L), indicating increased EC activity in the absence of Klf9. Furthermore, collagen IV staining revealed fewer signs of vascular regression in *Klf9^ΔEC^* retinas, characterized by fewer empty sleeves (Fig. 2M, N). Immunostaining for the pericyte marker, NG2, revealed a significant increase in pericyte coverage at the angiogenic sprouting front (Fig. 2O, P). Taken together, these results indicate that the loss of Klf9 in ECs impairs normal vascular pruning and vessel remodeling.

At P12, we assessed the vascular density in the superficial and deep retinal vascular plexuses. Although no significant differences were observed in the superficial plexus (Fig. 2Q, R), the deep vascular plexus showed a significant increase in vascular density in *Klf9^ΔEC^* mice compared with controls (Fig. 2Q, S). At P21, both superficial and deep plexuses in *Klf9^ΔEC^* mice exhibited the same level of vascular density (Fig. 2T-V). Collectively, these findings reveal that Klf9 functions as a temporal brake on angiogenesis, and the loss of endothelial Klf9 releases this brake, leading to accelerated early vascular expansion, delayed regression, and transiently increased vascular density.

### Endothelial-specific overexpression of Klf9 retards neonatal retinal vascular development

**Figure 3:**
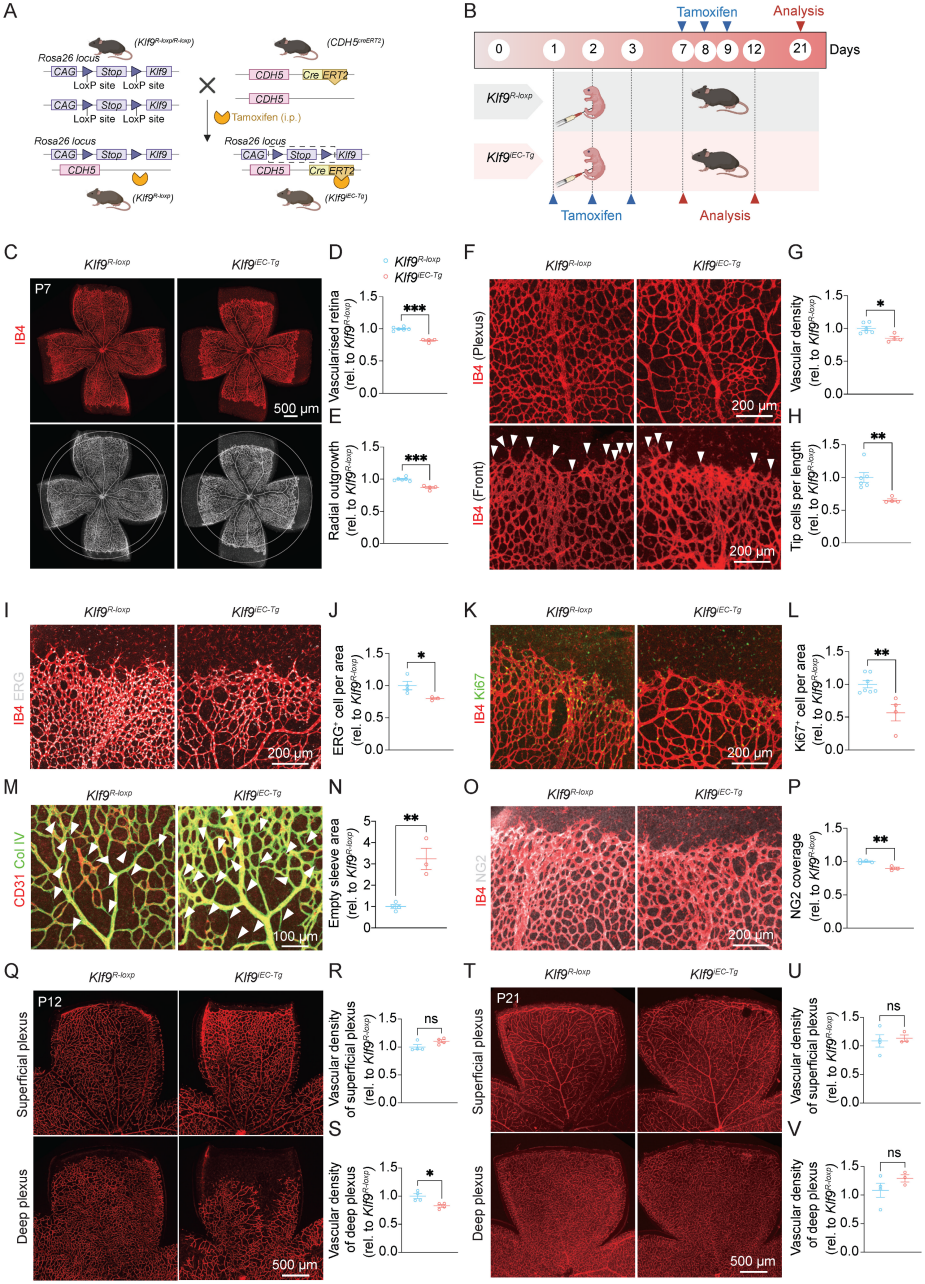
** Endothelial cell overexpression of Klf9 retards neonatal retinal vascular development*.*** (A) Schematic diagram of the generation of *Klf9^iEC-Tg^* mice. A tamoxifen-inducible endothelial-specific overexpression system was established by crossing *Cdh5(PAC)-CreERT2* mice with *Rosa26-LoxP-STOP-LoxP-Klf9* mice. (B) Schematic timeline showing tamoxifen administration from P1 to P3 with retinal analyses conducted at P7 and P12, or tamoxifen administration from P7 to P9 followed by analysis at P21. (C) Representative images of retinal flat mounts from *Klf9^R-loxp^* and *Klf9^iEC-Tg^* mice at P7, labeled with IB4 (red) to visualize the retinal vasculature. (D, E) Quantification of retinal vascularized area and radial outgrowth in *Klf9^R-loxp^* (n = 6) and *Klf9^iEC-Tg^* (n = 4) mice at P7. ***p < 0.001, mean ± SEM, two-tailed unpaired Student's t test. (F) High-magnification images of the vascular front showing vessel density and tip cells (arrowheads) in *Klf9^R-loxp^* and *Klf9^iEC-Tg^* retinas. (G, H) Quantification of vascular density and tip cell number per field in *Klf9^R-loxp^* (n = 6) and *Klf9^iEC-Tg^* (n = 4) mice at P7. *p < 0.05, **p < 0.01, mean ± SEM, two-tailed unpaired Student's t test. (I, J) Co-immunostaining and quantification of retinal vasculature (IB4, red) and endothelial nuclei (ERG, gray) in *Klf9^R-loxp^* (n = 4) and *Klf9^iEC-Tg^* (n = 3) retinas. *p < 0.05, mean ± SEM, two-tailed unpaired Student's t test. (K, L) Co-staining of proliferative marker Ki67 (green) and vasculature (IB4, red) in *Klf9^R-loxp^* (n = 7) and *Klf9^iEC-Tg^* (n = 4) retinas. **p < 0.01, mean ± SEM, two-tailed unpaired Student's t test. (M, N) Co-immunostaining and quantification for collagen IV (Col IV) (green) and IB4 (red) revealing vascular regression status (arrowheads) in *Klf9^R-loxp^* (n = 4) and *Klf9^iEC-Tg^* (n = 3) retinas. **p < 0.01, mean ± SEM, two-tailed unpaired Student's t test. (O, P) Co-immunostaining of retinal vasculature (IB4, red) with NG2 (grey) indicating pericyte coverage in *Klf9^R-loxp^* (n = 4) and *Klf9^iEC-Tg^* (n = 3) retinas; quantification shown in (P). **p < 0.01, mean ± SEM, two-tailed unpaired Student's t test. (Q-S) Representative images of superficial and deep vascular plexuses at P12 with quantifications of vascular density in* Klf9^R-loxp^* (n = 4) and *Klf9^iEC-Tg^* (n = 4) mice. *p < 0.05, mean ± SEM, two-tailed unpaired Student's t test. (T-V) Representative images and quantifications of superficial and deep vascular plexuses at P21 in* Klf9^R-loxp^* (n = 4) and *Klf9^iEC-Tg^* (n = 3) mice. Mean ± SEM, two-tailed unpaired Student's t test.

To confirm the role of EC-derived Klf9 in retinal vascular development, we generated inducible EC-specific Klf9 transgenic mice (*Klf9^iEC-Tg^*) and examined the effects of Klf9 overexpression on vascular formation (Fig. 3A). Similarly, we compared the retinal vasculature of *Klf9^iEC-Tg^* mice with that of *Klf9^R-loxp^* control mice at P7, P12, and P21 (Fig. 3B). RT-qPCR was performed to determine the efficiency of Klf9 overexpression in mouse retinal ECs (Supplementary Fig. 1C). Quantification of the vascularized area at P7 revealed a significant reduction in retinal vascularization in *Klf9^iEC-Tg^* mice compared to *Klf9^R-loxp^* controls, with *Klf9^iEC-Tg^* retinas exhibiting smaller vascular areas (Fig. 3C, D). Additionally, the radial extension of the vasculature was significantly reduced in *Klf9^iEC-Tg^* mouse retinas, indicating a delay in vascular growth (Fig. 3E). Staining of the retinal vasculature further demonstrated decreased vessel density and fewer tip cells in *Klf9^iEC-Tg^* mice than in controls (Fig. 3F-H). Immunohistochemical staining for the endothelial nucleus marker, ERG, showed a comparable decrease in the distribution of ECs in *Klf9^iEC-Tg^* mice (Fig. 3I, J). Additionally, co-immunostaining for the proliferative marker Ki67 with vessels revealed a significant reduction in cell proliferation within the retinal vasculature of *Klf9^iEC-Tg^* mice compared to controls (Fig. 3K, L). In addition, collagen IV staining revealed more signs of vascular regression in *Klf9^iEC-Tg^* retinas, which were characterized by more empty sleeves (Fig. 3M, N). Furthermore, immunostaining for the pericyte marker, NG2, revealed a significant decrease in pericyte coverage at the angiogenic sprouting front (Fig. 3O, P).

At P12, analysis of the retinal vasculature revealed defects in the deep vascular plexus in *Klf9^iEC-Tg^* mice compared with *Klf9^R-loxp^* controls, with a reduction in vascular density in the deep layer (Fig. 3Q-S). At P21, no significant differences in vascular density were observed in either the superficial or deep vascular plexuses between *Klf9^iEC-Tg^* and *Klf9^R-loxp^* mice (Fig. 3T-V). Collectively, these results support Klf9's role as a molecular brake that inhibits angiogenic growth. Accordingly, its overexpression suppresses vascularization, enhances regression, and decreases early vascular density.

### Klf9 expression is decreased in ocular neovascular diseases

**Figure 4:**
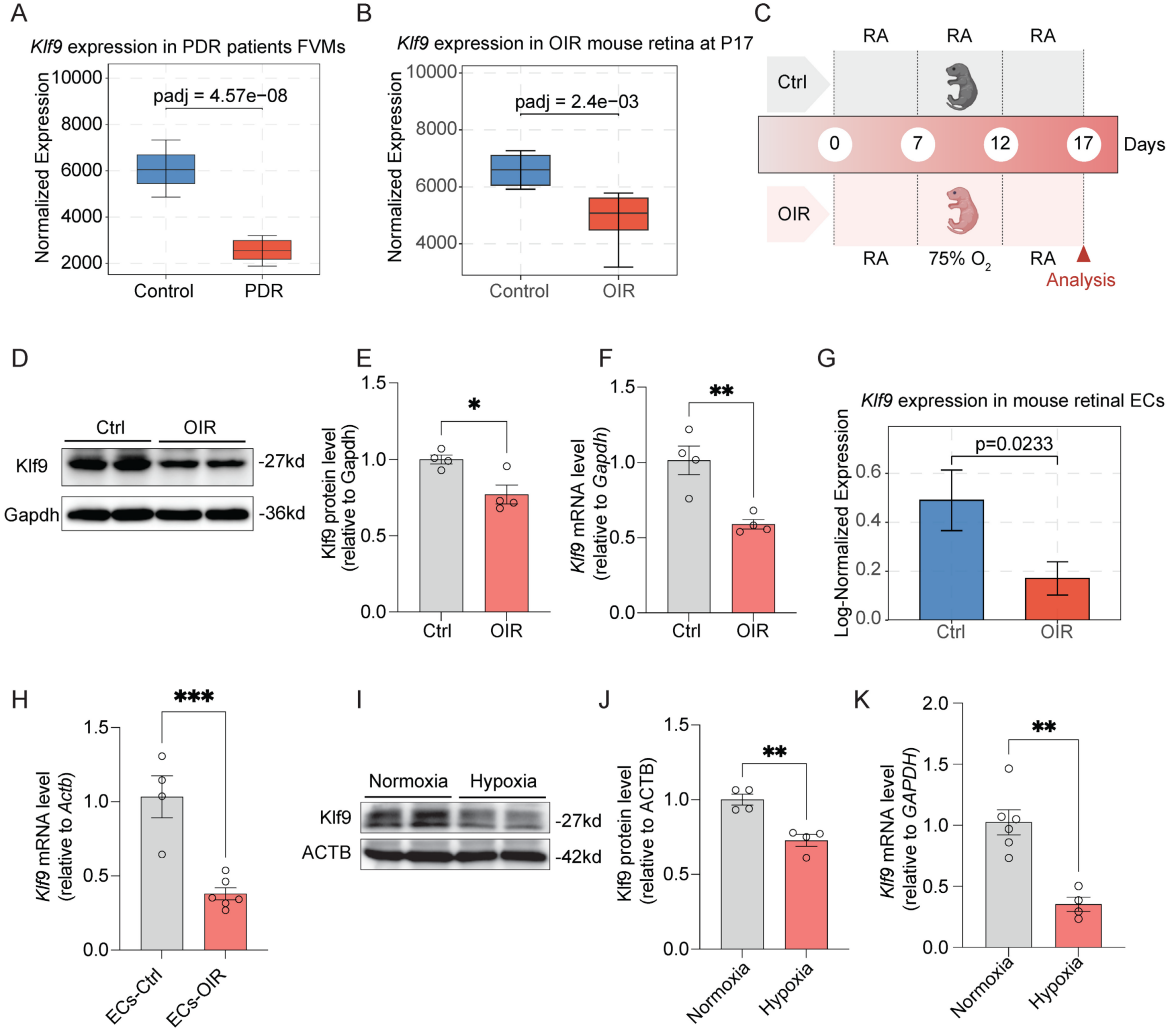
** Klf9 decreased in ocular neovascular diseases.** (A) Box plot showing Klf9 expression in the FVMs of PDR patients (data from GSE60436). Data are represented as normalized counts from the DESeq2 analysis. (B) Box plot displaying Klf9 expression in the OIR mouse retina at P17 (data from GSE234447). (C) Schematic diagram showing the experimental timeline of the oxygen-induced retinopathy (OIR) mouse model. (D) Western blotting showing protein expression of Klf9 and Gapdh in control and OIR mouse retinas at P17. (E) Quantification of Klf9 protein levels relative to Gapdh in control (n = 4) and OIR (n = 4) mouse retinas at P17. *p < 0.05, mean ± SEM, two-tailed unpaired Student's t test. (F) Quantitative RT-qPCR analysis of *Klf9* mRNA levels relative to *Gapdh* in the OIR (n = 4) and control (n = 4) retinas. **p < 0.01, mean ± SEM, two-tailed unpaired Student's t test. (G) Bar graph showing *Klf9* expression levels in retinal ECs from control and OIR mice (data from GSE150703). (H) Quantification of *Klf9* mRNA levels relative to *Gapdh* in mouse retinal ECs from control (n = 4) and OIR (n = 6) mice. ***p < 0.001, mean ± SEM, two-tailed unpaired Student's t test. (I) Western blotting analysis of Klf9 expression in HRMECs (human retinal microvascular endothelial cells) under normoxic and hypoxic conditions. (J) Quantification of Klf9 protein levels in HRMECs under normoxic (n = 4) and hypoxic (n = 4) conditions. **p < 0.01, mean ± SEM, two-tailed unpaired Student's t test. (K) Quantification of *Klf9* mRNA levels relative to *GAPDH* in HRMECs exposed to normoxia (n = 6 biological replicates) or hypoxia (n = 4 biological replicates). **p < 0.01, mean ± SEM, two-tailed unpaired Student's t test.

To investigate the role of Klf9 in ocular neovascular diseases, we analyzed its expression in patients with PDR and in OIR mice. Box plot analysis of data from GSE60436 36 revealed a significant decrease in Klf9 expression in the fibrovascular membranes of patients with PDR compared to that in the control group (Fig. 4A). In the OIR mouse model, retinal Klf9 expression was also significantly reduced compared with control mice at P17 (data from GSE234447 32) (Fig. 4B). To validate these findings, we constructed an OIR mouse model according to standard procedures (Fig. 4C) and performed western blot analysis of retinal extracts from control and OIR mice at P17 (Fig. 4D). Western blot analysis revealed significantly reduced Klf9 protein levels in OIR retinas (Fig. 4E), consistent with the significant decrease in *Klf9* mRNA levels measured by RT-qPCR (Fig. 4F).

We further investigated the expression of Klf9 in mouse retinal ECs, given its critical role in retinal neovascularization. According to single-cell sequencing data (GSE150703) 34, Klf9 expression was significantly reduced in OIR mouse retinal ECs compared to that in control mice (Fig. 4G). We isolated CD31-positive mouse retinal ECs using magnetic bead sorting and subjected them to RT-qPCR. RT-qPCR results confirmed that *Klf9* mRNA levels were significantly decreased in ECs from OIR mouse retinas compared to control mice (Fig. 4H).

As hypoxia is the main driver of pathological angiogenesis in conditions such as PDR and the OIR model recapitulates hypoxia-driven angiogenesis, we further explored the effect of hypoxia on Klf9 expression in HRMECs. Western blot analysis revealed that Klf9 protein levels were significantly reduced under hypoxia compared to those under normoxia (Fig. 4I, J). Similarly, *Klf9* mRNA levels were significantly lower in HRMECs exposed to hypoxia than those exposed to normoxia (Fig. 4K). Collectively, these data verify that Klf9 is significantly downregulated in retinal vascular ECs in the context of pathological retinal neovascularization.

### Endothelial-specific deletion of Klf9 aggravates pathological retinal neovascularization

**Figure 5:**
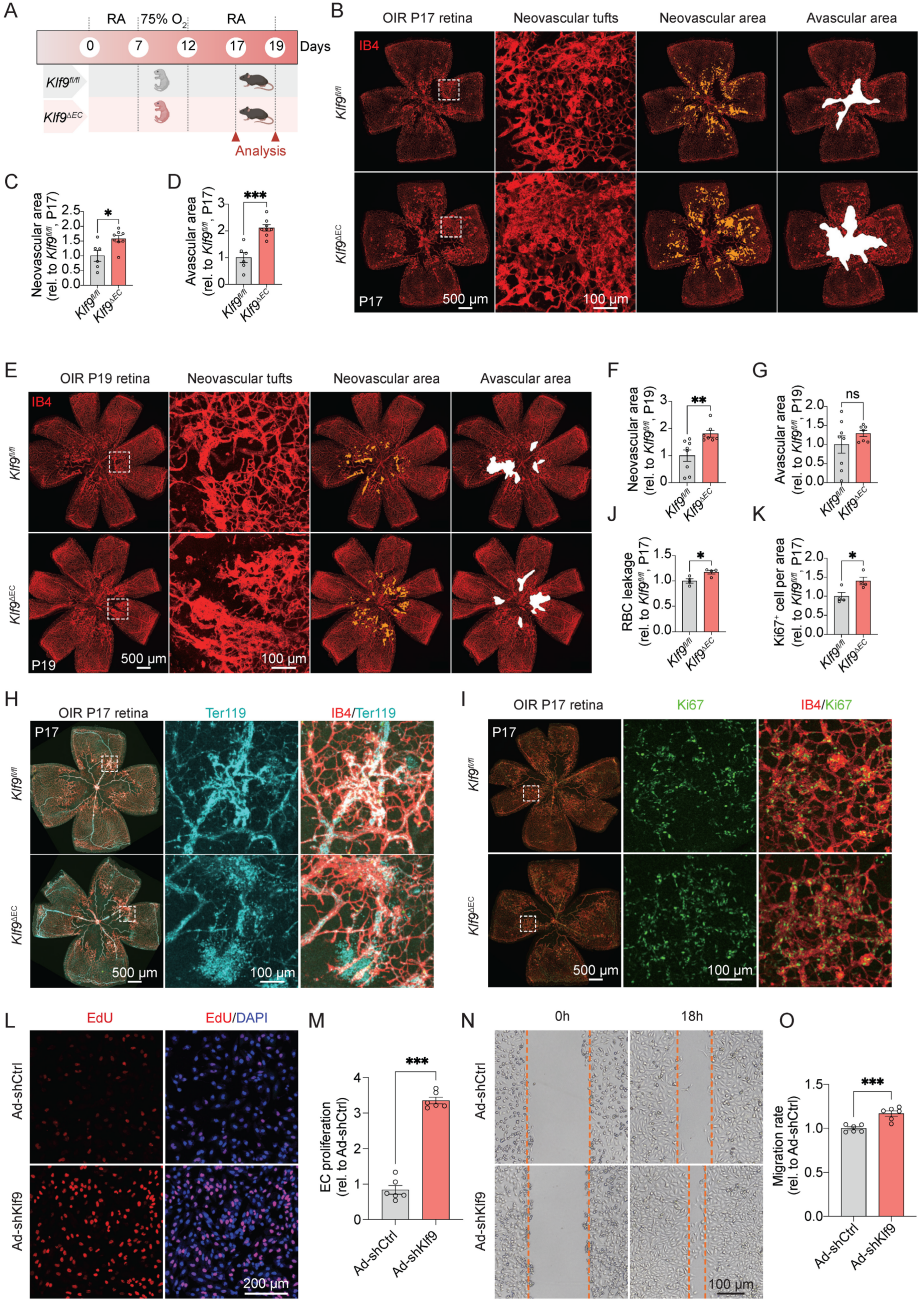
** Klf9 deficiency in ECs aggravates oxygen-induced retinopathy.** (A) Schematic diagram and experimental design of the oxygen-induced retinopathy (OIR) model in *Klf9^fl/fl^* and *Klf9^∆EC^* mice. (B) Representative images of retinal flat mounts from OIR mice at P17. IB4 (red) was used to visualize the vasculature, showing neovascular tufts (yellow) and avascular areas (white) in *Klf9^fl/fl^* and *Klf9^∆EC^* mice. (C-D) Quantification of the neovascular area and avascular area in *Klf9^fl/fl^* (n = 6) and *Klf9^∆EC^* (n = 8) mice at P17. *p < 0.05, ***p < 0.001, mean ± SEM, two-tailed unpaired Student's t test. (E) Representative images of retinal flat mounts from OIR mice at P19 in *Klf9^fl/fl^* and *Klf9^∆EC^* mice. (F-G) Quantification of the neovascular area and avascular area in *Klf9^fl/fl^* (n = 8) and *Klf9^∆EC^* (n = 7) mice at P19. **p < 0.01, mean ± SEM, two-tailed unpaired Student's t test. (H, J) Co-immunostaining and quantification of IB4 (red) and Ter119 (cyan) to assess vascular leakage in OIR retinas from *Klf9^fl/fl^* (n = 4) and *Klf9^∆EC^* (n = 4) at P17. *p < 0.05, mean ± SEM, two-tailed unpaired Student's t test. (I, K) Co-immunostaining and quantification of IB4 (red) and the proliferation marker Ki67 (green) to evaluate EC proliferation in OIR retinas from *Klf9^fl/fl^* (n = 4) and *Klf9^∆EC^* (n = 4) at P17. *p < 0.05, mean ± SEM, two-tailed unpaired Student's t test. (L) Representative images of EdU incorporation (red) in HRMECs infected with Ad-shCtrl and Ad-shKlf9. (M) Quantification of the percentage of EdU-positive cells in HRMECs infected with Ad-shCtrl (n = 6 biological replicates) and Ad-shKlf9 (n = 6 biological replicates). ***p < 0.001, mean ± SEM, two-tailed unpaired Student's t test. (N) Representative images of scratch wound assays showing migration of HRMECs infected with Ad-shCtrl and Ad-shKlf9 at 0 h and 18 h. (O) Quantification of the migration rate in HRMECs infected with Ad-shCtrl (n = 6 biological replicates) and Ad-shKlf9 (n = 6 biological replicates) at 18 h. ***p < 0.001, mean ± SEM, two-tailed unpaired Student's t test.

To evaluate the role of EC-specific Klf9 deficiency in retinal neovascularization, we constructed the OIR model in *Klf9^fl/fl^* and *Klf9^∆EC^* mice to assess the pathological outcomes at P17 and P19 (Fig. 5A). The OIR model captures two key disease stages: P17 represents the peak pathological neovascularization, mimicking the proliferative phase of retinopathy, where senescent endothelial cells accumulate 37; and P19 marks the onset of vascular regression, where neutrophil-mediated clearance of diseased vessels promotes physiological remodeling 34. Retinal flat mounts from *Klf9^fl/fl^* and *Klf9^∆EC^* mice at P17 showed that *Klf9^∆EC^* mice exhibited increased neovascular tuft formation and avascular area compared with *Klf9^fl/fl^* controls (Fig. 5B-D). At P19, the retinal neovascular area of *Klf9^∆EC^* mice also increased compared with controls (Fig. 5E-G). Co-immunostaining for IB4 and Ter119 was performed at P17 to assess retinal hemorrhage, and the data revealed increased red blood cell (RBC) leakage in *Klf9^∆EC^* retinas compared with *Klf9^fl/fl^* controls (Fig. 5H, J). Furthermore, co-immunostaining for IB4 and Ki67 displayed enhanced EC proliferation in *Klf9^∆EC^* mice compared with controls, suggesting that the absence of Klf9 in ECs leads to increased EC proliferation in OIR retinas (Fig. 5I, K).

To further confirm the role of Klf9 in ECs *in vitro*, we transduced HRMECs with an adenovirus to knockdown Klf9 expression (Supplementary Fig. 2A-C) and assessed subsequent functional alterations. The EdU incorporation assay was performed to assess cell proliferation. Representative images of EdU incorporation in HRMECs showed increased proliferation in the Klf9-deficient group compared to control cells (Fig. 5L, M). EC migration was evaluated using scratch wound assay. Representative images of the wound closure at 0h and 18h showed increased migration in Klf9-deficient HRMECs compared to controls, indicating that Klf9 deficiency enhanced EC migration (Fig. 5N, O). Collectively, these findings suggest that Klf9 deficiency in ECs exacerbates the progression of pathological retinal neovascularization by hyperactivating ECs.

### Endothelial-specific overexpression of Klf9 ameliorates pathological retinal neovascularization progression

**Figure 6:**
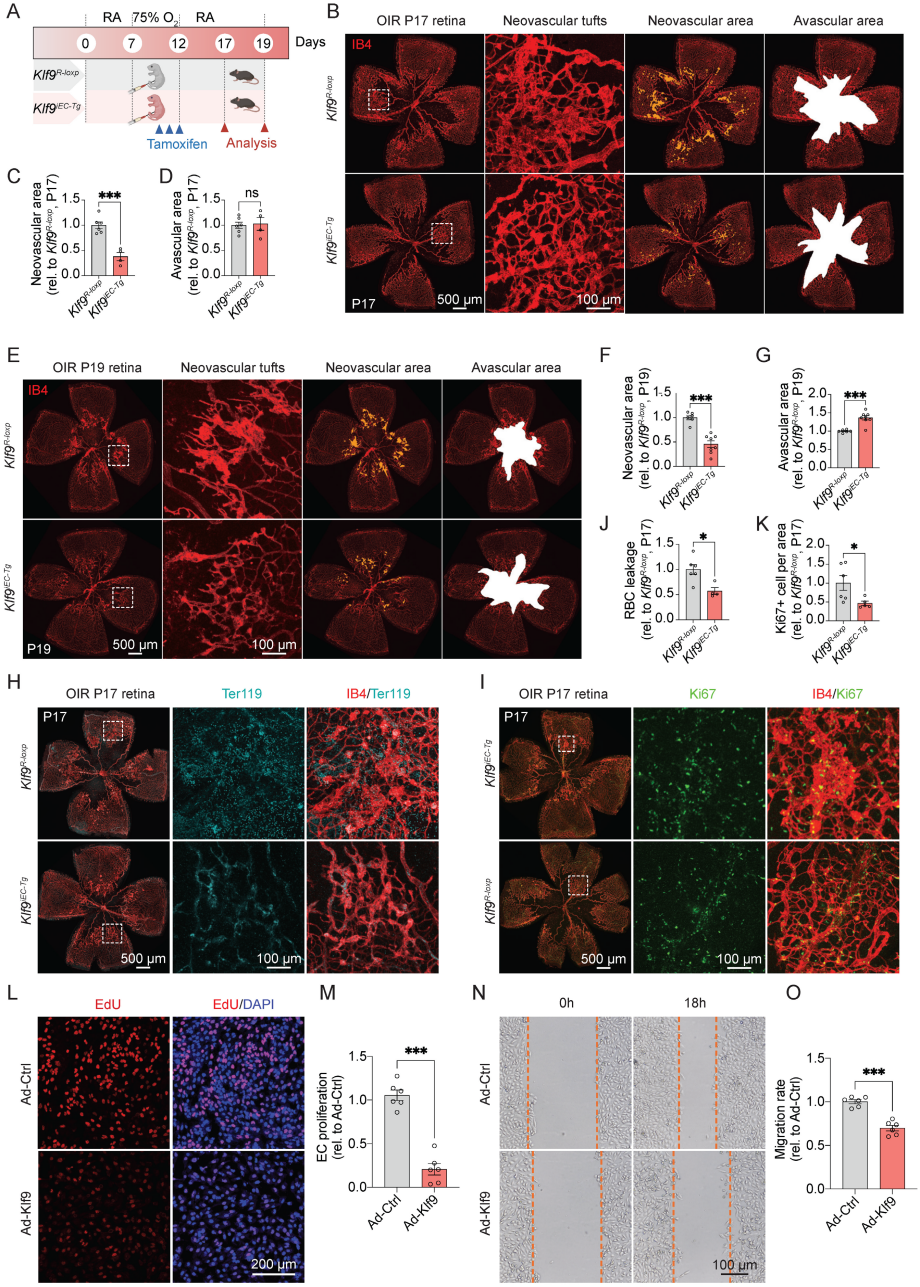
** Endothelial cell overexpression of Klf9 ameliorates OIR progression.** (A) Schematic diagram and experimental design of the oxygen-induced retinopathy (OIR) model in *Klf9^R-loxp^* and *Klf9^iEC-Tg^* mice. (B) Representative images of retinal flat mounts from OIR mice showing neovascular tufts (yellow) and avascular areas (white) in *Klf9^R-loxp^* and *Klf9^iEC-Tg^* mice at P17. (C-D) Quantification of the neovascular area and avascular area in *Klf9^R-loxp^* (n = 6) and *Klf9^iEC-Tg^* (n = 4) mice at P17. ***p < 0.001, mean ± SEM, two-tailed unpaired Student's t test. (E) Representative images of retinal flat mounts from OIR mice in *Klf9^R-loxp^* and *Klf9^iEC-Tg^* mice at P19. (F-G) Quantification of the neovascular area and avascular area in *Klf9^R-loxp^* (n = 6) and *Klf9^iEC-Tg^* (n = 8) mice at P19. ***p < 0.001, mean ± SEM, two-tailed unpaired Student's t test. (H, J) Co-immunostaining and quantification of retinal vasculature (IB4, red) and Ter119 (cyan) to visualize vascular leakage in OIR retinas from *Klf9^R-loxp^* (n = 6) and *Klf9^iEC-Tg^* (n = 4) mice. *p < 0.05, ***p < 0.001, mean ± SEM, two-tailed unpaired Student's t test. (I, K) Co-immunostaining and quantification of retinal vasculature (IB4, red) and the proliferation marker Ki67 (green) to assess EC proliferation in OIR retinas from *Klf9^R-loxp^* (n = 6) and *Klf9^iEC-Tg^* (n = 5) mice. *p < 0.05, ***p < 0.001, mean ± SEM, two-tailed unpaired Student's t test. (L) Representative images of EdU incorporation (red) in HRMECs infected with Ad-Ctrl and Ad-Klf9. (M) Quantification of proliferation in HRMECs infected with Ad-Ctrl (n = 6 biological replicates) and Ad-Klf9 (n = 6 biological replicates). ***p < 0.001, mean ± SEM, two-tailed unpaired Student's t test. (N) Representative images of scratch wound assays in HRMECs infected with Ad-Ctrl and Ad-Klf9 at 0 h and 18 h. (O) Quantification of migration rate in HRMECs infected with Ad-Ctrl (n = 6 biological replicates) and Ad-Klf9 (n = 6 biological replicates) at 18h. ***p < 0.001, mean ± SEM, two-tailed unpaired Student's t test.

To evaluate the therapeutic potential of Klf9 overexpression, we induced OIR in *Klf9^iEC-Tg^* mice and assessed its effects on pathological angiogenesis (Fig. 6A). Representative images of the retinal vasculature and corresponding quantification revealed a marked reduction in neovascular tuft formation in *Klf9^iEC-Tg^* mice compared with controls (Fig. 6B-D). Consistently, the neovascular area of *Klf9^iEC-Tg^* mice also decreased at P19 compared with controls (Fig. 6E-G). Furthermore, the RBC leakage area at P17 in *Klf9^iEC-Tg^* mice was significantly decreased compared with *Klf9^R-loxp^* mice (Fig. 6H, J). *Klf9^iEC-Tg^* mice displayed decreased EC proliferation with fewer Ki67-positive ECs in the retina at P17 compared with controls, suggesting that Klf9 overexpression ameliorated EC proliferation in OIR retinas (Fig. 6I, K).

Similarly, we transduced HRMECs with an adenovirus to overexpress Klf9 *in vitro* (Supplementary Fig. 2D-F) and assessed subsequent functional changes. The EdU incorporation assay showed decreased proliferation in cells overexpressing Klf9 compared with controls (Fig. 6L, M). The scratch wound assay verified that Klf9 overexpression significantly reduced EC migration at 18 h (Fig. 6N, O). Collectively, these findings suggest that EC overexpression in Klf9 ameliorates pathological retinal neovascularization by decreasing EC proliferation, migration, and neovascular tuft formation.

### Klf9 regulates the expression of genes related to angiogenesis and cell adhesion in HRMECs

**Figure 7:**
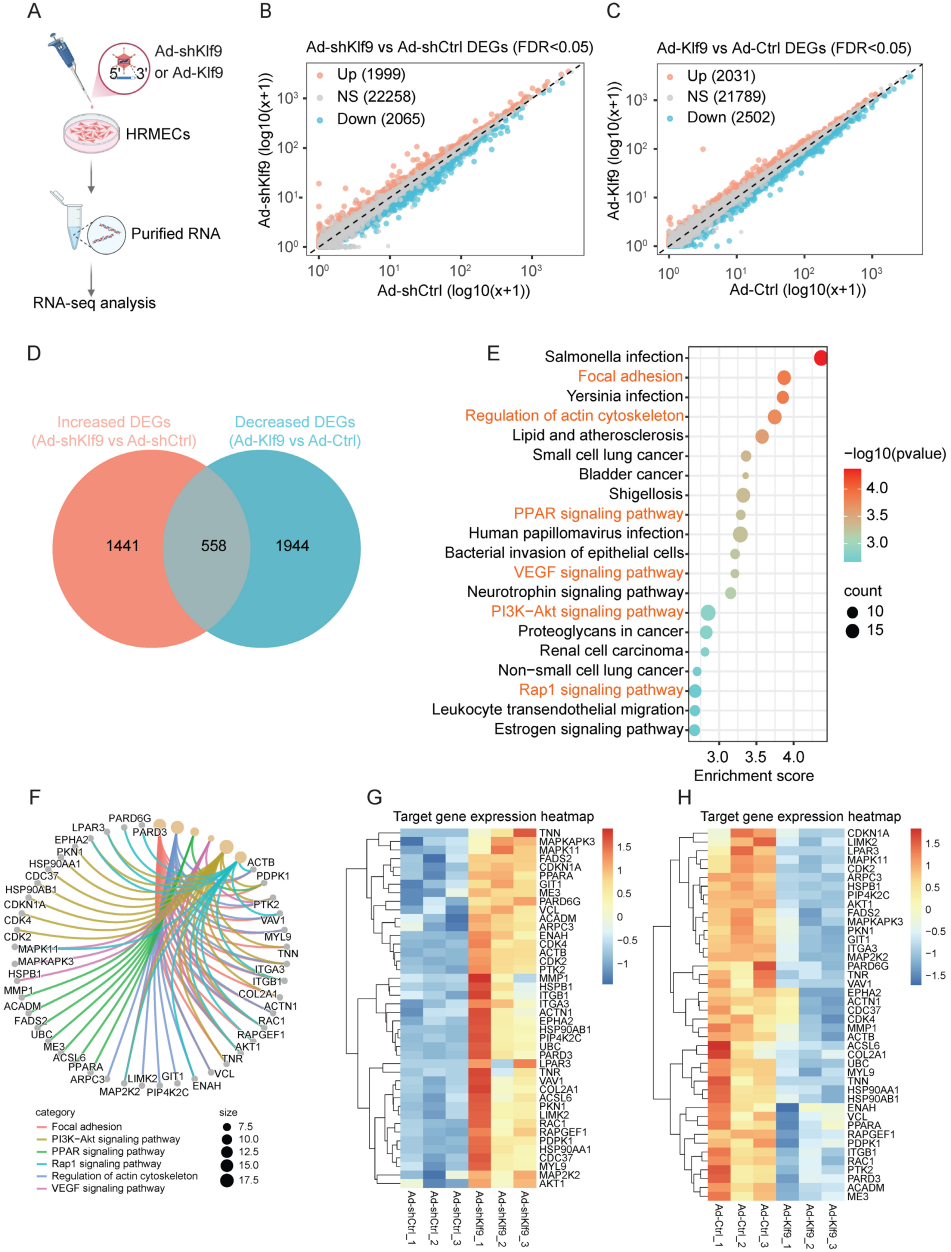
** Transcriptomic analysis of Klf9 knockdown and overexpression in HRMECs.** (A) Schematic of the experimental workflow. HRMECs were transduced with Ad-shKlf9 or Ad-Klf9 and their control adenovirus (n = 3 biological replicates per group) followed by RNA isolation and RNA-seq analysis. (B) Scatter plot of differentially expressed genes (DEGs) between Ad-shKlf9 and Ad-shCtrl groups. Genes significantly upregulated (red), downregulated (blue), or unchanged (gray) are indicated. (C) Scatter plot of DEGs between Ad-Klf9 and Ad-Ctrl groups. Genes significantly upregulated (red), downregulated (blue), and unchanged (gray) are indicated. (D) Venn diagram showing the overlap between increased DEGs in the Ad-shKlf9 group and decreased DEGs in the Ad-Klf9 group. (E) KEGG pathway enrichment analysis of overlapping DEGs. Pathways are ranked by enrichment score, with circle size representing gene count and color scale representing statistical significance (-log10 (p-value)). (F) Chord diagram showing key DEGs involved in focal adhesion, PI3K-Akt signaling, PPAR signaling, Rap1 signaling, actin cytoskeleton regulation, and VEGF signaling pathways. (G-H) Heatmaps of representative target genes in the enriched pathways. Gene expression is shown as row-normalized Z-scores across replicates in the Ad-shKlf9 vs Ad-shCtrl (G) and Ad-Klf9 vs Ad-Ctrl (H) groups.

To explore the transcriptional roles and downstream effects of Klf9 in ECs, we performed RNA sequencing following Klf9 knockdown (Ad-shKlf9) and overexpression (Ad-Klf9) in HRMECs (Fig. 7A; Supplementary Fig. 3A, B). Differential expression analysis revealed that 1,999 genes were upregulated, and 2,065 genes were downregulated in the Ad-shKlf9 group (Fig. 7B). Conversely, 2,031 genes were upregulated, and 2,502 genes were downregulated in the Ad-Klf9 group (Fig. 7C). A comparison of the altered genes showed that 558 genes were inversely regulated between knockdown and overexpression conditions, and these genes may represent key Klf9-dependent targets (Fig. 7D).

KEGG Pathway enrichment analysis of the overlapping genes revealed the involvement of several critical signaling pathways, including focal adhesion, PI3K-Akt, PPAR, Rap1, VEGF signaling, and actin cytoskeleton regulation, which are essential for vessel function (Fig. 7E). A chord diagram delineates the involvement of specific genes in these pathways (Fig. 7F). The involvement of several genes, such as *AKT1, PTK2, RAC1, MYL9, RAPGEF1,* and* VCL*, across multiple pathways indicates their pivotal roles in the regulatory network. Serving as the central signaling hub downstream of VEGF, the PI3K/Akt pathway propels angiogenesis by activating RAC1 for focal adhesion-driven migration, recruiting Rap1 to stabilize adhesions and junctions, and modulating metabolic and inflammatory responses via PPAR signaling 38, 39. Previous studies have confirmed that the PI3K/Akt pathway is indispensable for EC functions, including cell proliferation, migration, and vascular tone 24, 40. Notably, conditional deletion of Akt1 rather than Akt2 in ECs retards mouse retinal angiogenesis at P7, implying that Akt1 exerts a non-redundant function during physiological angiogenesis 24. In addition, focal adhesions, which are dynamic structures that link the extracellular matrix to the actin cytoskeleton, also serve as critical signaling hubs in angiogenesis 41, 42. Generally, VEGF activation promotes the VEGFR3-dependent PI3K/AKT activation and subsequent recruitment of RAC1 to nascent focal adhesions, where active RAC1 locally drives actin polymerization to extend lamellipodia, propelling directional migration during vascular sprouting 42, 43. Moreover, the clustering heatmap visualized specific shifts in gene expression levels within the key KEGG-enriched pathways across the Ad-shKlf9, Ad-shCtrl, Ad-Klf9, and Ad-Ctrl samples (Fig. 7G, H).

Collectively, these results identified Klf9 as a critical transcriptional regulator in ECs that governs the major signaling networks related to angiogenesis and cell adhesion. This function underscores their pivotal role in preserving endothelial homeostasis and ensuring proper vascular development and integrity.

### Klf9 suppresses the chromatin accessibility of PI3K-Akt signaling pathway and adhesion-related pathway genes

**Figure 8:**
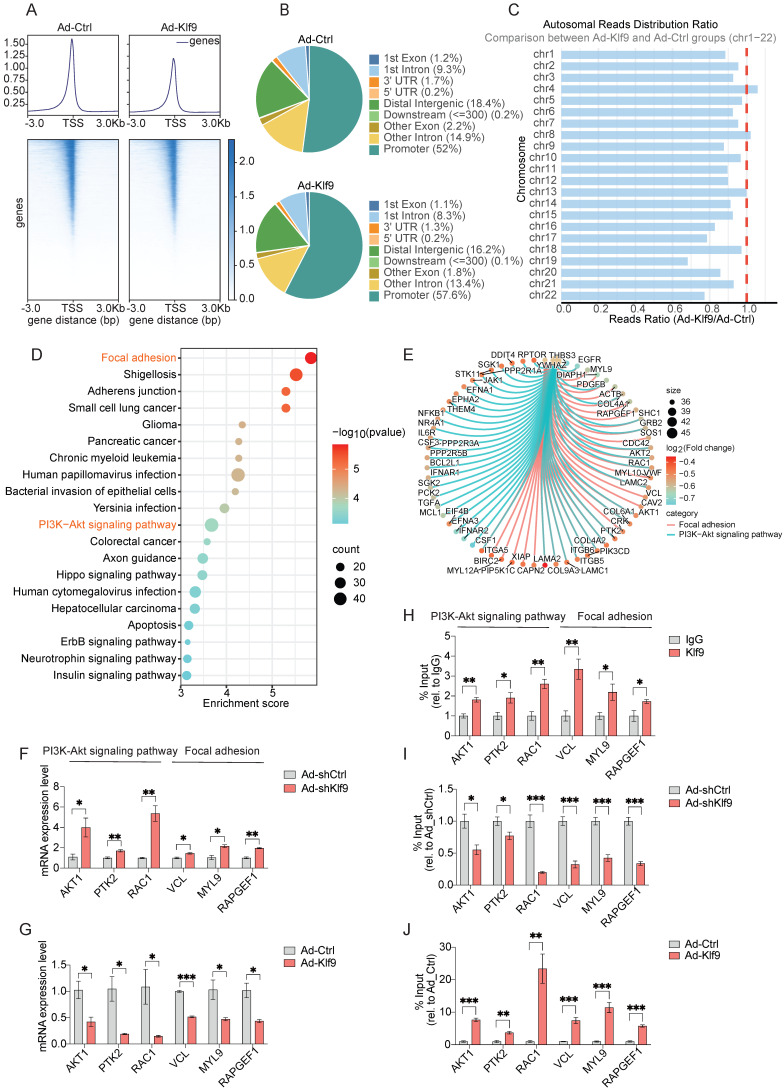
** Klf9 suppresses chromatin accessibility at the promoters of PI3K-Akt signaling pathway- and focal adhesion-related genes.** (A) Distribution of sequencing reads around transcription start sites (TSS) in control (Ad-Ctrl) and Klf9-overexpressing (Ad-Klf9) HRMECs. Average profiles (top) and heatmaps (bottom) are shown within ±3 kb of TSS. (B) Genomic annotation of chromatin accessibility peaks identified by ATAC-seq in Ad-Ctrl and Ad-Klf9 groups. Pie charts display the distribution of accessible regions across genomic features. (C) Chromosomal distribution of peaks across autosomes (chr1-22). The ratio of reads in Ad-Klf9 relative to Ad-Ctrl is shown. (D) KEGG pathway enrichment analysis of down-regulated differentially accessible regions (DARs). Top significantly enriched pathways are shown, with dot size indicating gene count and color scale representing statistical significance (-log10 (p-value)). (E) Chord diagram showing representative genes enriched in focal adhesion and PI3K-Akt signaling pathways. Genes are colored based on log2 fold change, and node size reflects the number of associated interactions. (F-G) Validation of representative target gene expression by qPCR in HRMECs following Klf9 knockdown (Ad-shKlf9 vs Ad-shCtrl, n = 3 per biological replicates group) or Klf9 overexpression (Ad-Klf9 vs Ad-Ctrl, n = 3 per biological replicates group). *p < 0.05, **p < 0.01, ***p < 0.001, mean ± SEM, two-tailed unpaired Student's t test. (H-J) ChIP-qPCR validation of Klf9 binding to target gene promoters in HRMECs after Klf9 knockdown (Ad-shKlf9 vs Ad-shCtrl, n = 4 biological replicates per group) or Klf9 overexpression (Ad-Klf9 vs Ad-Ctrl, n = 4 biological replicates per group). *p < 0.05, **p < 0.01, ***p < 0.001, mean ± SEM, two-tailed unpaired Student's t test.

To determine the genomic binding features of Klf9 as a transcription factor, we performed ATAC-seq analysis of HRMECs after Klf9 overexpression. Metagene analysis showed that sequencing reads were highly enriched around TSS, indicating that Klf9 may play a pivotal role in regulating gene expression. Notably, we observed decreased chromatin accessibility and altered transcriptional regulation in the Ad-Klf9 group compared to those in the Ad-Ctrl group (Fig. 8A). This suggests that Klf9 overexpression leads to a more condensed chromatin structure, which can repress downstream target genes critical for endothelial function. Further analysis of the binding peaks revealed that approximately 58% of the peaks were located within the promoter regions, followed by the distal intergenic and intronic regions (Fig. 8B). Chromosomal distribution analysis indicated that chromatin accessibility broadly decreased across autosomes in the Ad-Klf9 group (Fig. 8C).

Functional enrichment of the downregulated differentially accessible regions identified multiple pathways related to endothelial biology, including focal adhesion and PI3K-Akt signaling (Fig. 8D). A chord diagram highlighted several hub genes, including *AKT1, PTK2, RAC1, MYL9, RAPGEF1 and VCL*, which are involved in both the PI3K-Akt pathways and focal adhesion (Fig. 8E). The results of the functional enrichment analysis of the ATAC-seq data were highly consistent with the RNA sequencing findings, demonstrating that pathways closely related to vascular function, including PI3K-Akt signaling and focal adhesion, are regulated by Klf9.

Based on the central roles of the PI3K/Akt pathway in angiogenesis and focal adhesions during EC migration, we performed RT-qPCR and chromatin immunoprecipitation followed by qPCR (ChIP-qPCR) to analyze representative genes identified by integrated RNA-seq and ATAC-seq. Klf9 knockdown significantly increased the expression of *AKT1, PTK2, RAC1, MYL9, RAPGEF1 and VCL*, whereas Klf9 overexpression decreased their expression (Fig. 8F-G). Further, ChIP-qPCR validation confirmed Klf9 occupancy at specific promoters of *AKT1, PTK2, RAC1, MYL9, RAPGEF1 and VCL* (Fig. 8H-J). These results suggested that Klf9 regulates a set of proliferation-, adhesion-, and migration-related genes, at least in part by modulating the PI3K-Akt signaling and focal adhesion pathways. Functionally, the PI3K-Akt pathway serves as a central signaling hub that integrates inputs from upstream signals such as growth factor receptors and focal adhesions to coordinate essential cellular decisions, including survival, proliferation, metabolism, and migration 38. Although focal adhesions are critical for spatial signaling initiation, PI3K-Akt acts as a key downstream convergence point and amplifier 39, 44. Its pivotal role renders it a principal target of regulatory genes such as Klf9. Consequently, elucidating Klf9 modulation of the PI3K-Akt pathway is essential to determine whether Klf9 executes its biological functions through the regulation of this central signaling axis, thereby exerting a broad influence on downstream cellular processes.

### Modulation of the PI3K-Akt signaling pathway rescues Klf9-induced EC dysfunction *in vitro*

**Figure 9:**
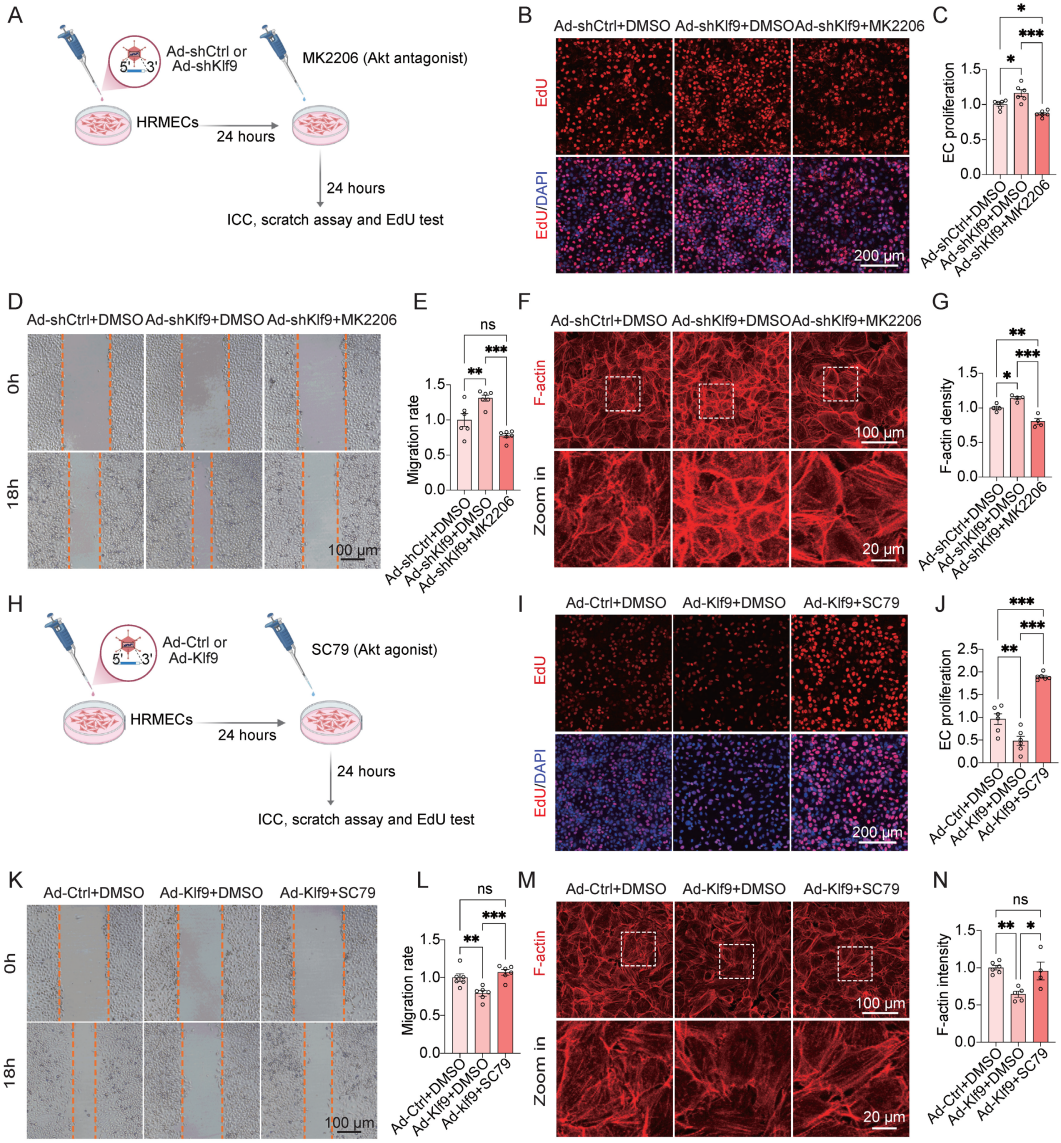
** Activation or inhibition of the PI3K-Akt signaling pathway rescues Klf9-induced EC dysfunction *in vitro*.** (A) Schematic diagram of the experimental timeline for Klf9 knockdown and subsequent Akt inhibition in HRMECs. (B, C) Representative fluorescence images and quantitative analysis of EdU incorporation assays assessing HRMEC proliferation in Ad-shCtrl + DMSO, Ad-shKlf9 + DMSO, Ad-shKlf9 + MK2206 groups (n = 6 biological replicates per group). *p < 0.05, ***p < 0.001, mean ± SEM, one-way ANOVA with Tukey's multiple comparisons test. (D, E) Representative images and quantification of scratch wound healing assays evaluating HRMEC migration in Ad-shCtrl + DMSO, Ad-shKlf9 + DMSO, Ad-shKlf9 + MK2206 groups (n = 6 biological replicates per group). **p < 0.01, ***p < 0.001, mean ± SEM, one-way ANOVA with Tukey's multiple comparisons test. (F, G) Representative F-actin immunofluorescence images and quantitative analysis of fluorescence area per cell in Ad-shCtrl + DMSO, Ad-shKlf9 + DMSO, Ad-shKlf9 + MK2206 groups (n = 4 biological replicates per group). *p < 0.05, **p < 0.01, ***p < 0.001, mean ± SEM, one-way ANOVA with Tukey's multiple comparisons test. (H) Schematic diagram of the experimental timeline for Klf9 overexpression and subsequent Akt activation in HRMECs. (I, J) Representative fluorescence images and quantitative analysis of EdU incorporation assays assessing HRMEC proliferation in Ad-Ctrl + DMSO, Ad-Klf9 + DMSO, Ad-Klf9 + SC79 groups (n = 6 biological replicates per group). **p < 0.01, ***p < 0.001, mean ± SEM, one-way ANOVA with Tukey's multiple comparisons test. (K, L) Representative images and quantification of scratch wound healing assays evaluating HRMEC migration in Ad-Ctrl + DMSO, Ad-Klf9 + DMSO, Ad-Klf9 + SC79 groups (n = 6 biological replicates per group). **p < 0.01, ***p < 0.001, mean ± SEM, one-way ANOVA with Tukey's multiple comparisons test. (M, N) Representative F-actin immunofluorescence images and quantitative analysis of fluorescence area per cell in Ad-Ctrl + DMSO, Ad-Klf9 + DMSO, Ad-Klf9 + SC79 groups (n = 6, 5, and 4 biological replicates, respectively). *p < 0.05, **p < 0.01, mean ± SEM, one-way ANOVA with Tukey's multiple comparisons test.

To investigate whether the PI3K-Akt signaling pathway mediates the regulatory effects of Klf9 on EC function, we performed a series of rescue experiments using pharmacological inhibition of Akt *in vitro* (Fig. 9A; Supplementary Fig. 4A, B). Functional assays confirmed that the promoting effects of Klf9 knockdown on EC migration and proliferation, as measured by EdU incorporation and scratch wound healing, were significantly suppressed by the Akt inhibitor MK2206 (Fig. 9B-E). Moreover, F-actin staining of the cytoskeletal organization revealed that Klf9 knockdown enhanced actin polymerization, and this effect was abolished by MK2206 (Fig. 9F-G).

Conversely, pharmacological activation of Akt was observed in HRMECs after infection with Ad-Klf9 and Ad-Ctrl (Fig. 9H; Supplementary Fig. 4C, D). Similarly, the impairment of EC migration and proliferation induced by Klf9 overexpression was rescued by treatment with the Akt agonist, SC79 (Fig. 9I-L). In addition, Klf9 overexpression reduced F-actin density, which was rescued by SC79 treatment (Fig. 9M, N). These findings indicate that Klf9 negatively regulates Akt activation and that its functional effects can be modulated by Akt modulators.

In summary, these rescue experiments provide functional evidence that Klf9 modulates EC migration, proliferation, and cytoskeletal organization, primarily through the PI3K-Akt signaling pathway. Genetic and pharmacological interventions have confirmed that Akt acts as a key downstream effector of Klf9 in regulating endothelial function.

### Modulation of the PI3K-Akt signaling pathway rescues Klf9-induced vasculature development disorder *in vivo*

**Figure 10:**
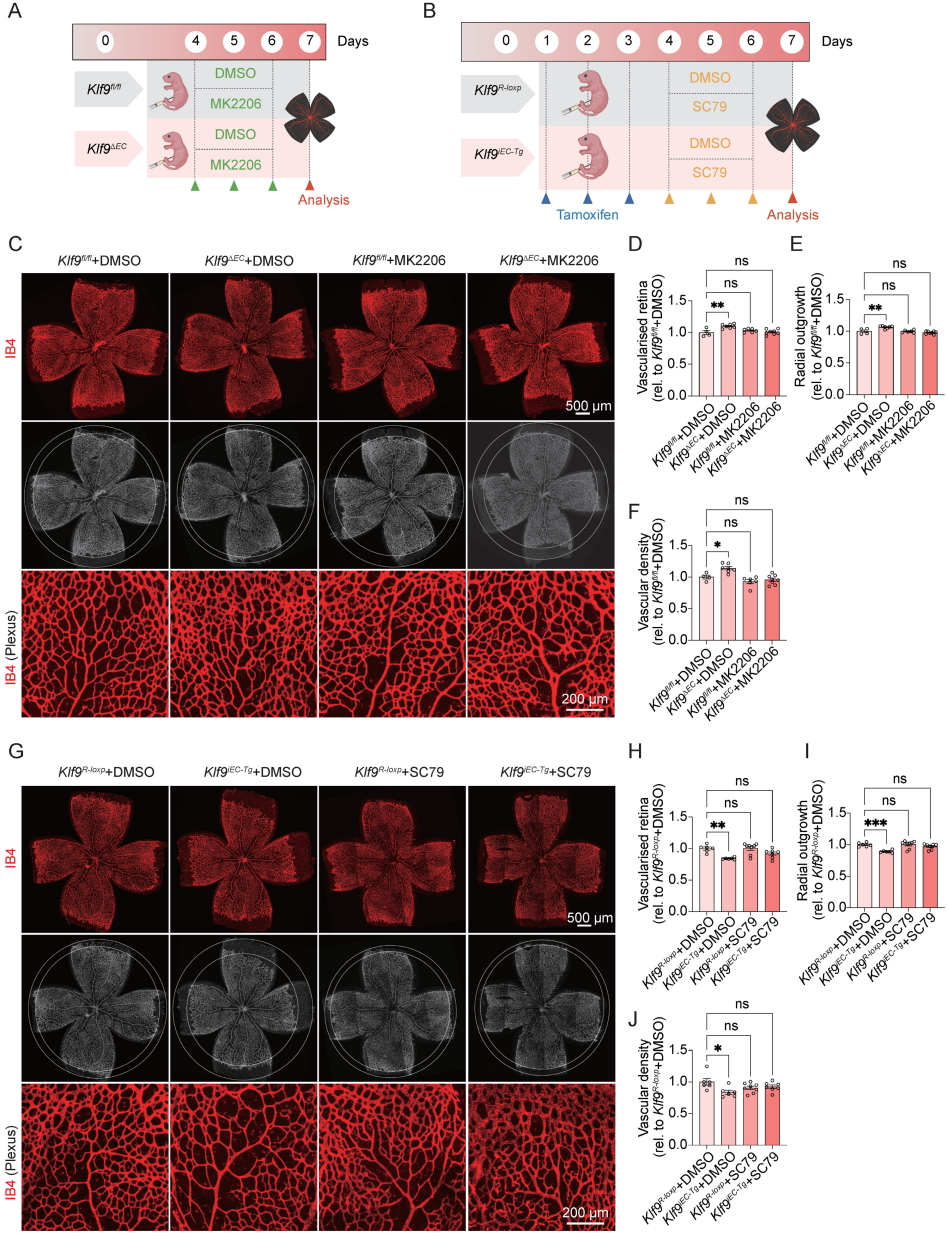
** Activation or inhibition of PI3K-Akt signaling pathway rescues Klf9-induced vascular development abnormalities *in vivo*.** (A) Experimental timeline for Akt antagonist (MK2206) administration in *Klf9^∆EC^* and *Klf9^fl/fl^* mice. (B) Experimental timeline for tamoxifen and Akt agonist (SC79) administration in *Klf9^iEC-Tg^* and *Klf9^R-loxp^* mice. (C-F) Representative whole-mount images and quantification of vascularized retinal area, radial outgrowth of P7 retinas from DMSO or MK2206 treated *Klf9^fl/fl^* (n = 4 for DMSO, n = 6 for MK2206) and *Klf9^∆EC^* (n = 7 for DMSO, n = 8 for MK2206) mice stained with IB4 (red, vasculature). *p < 0.05, **p < 0.01, mean ± SEM, one-way ANOVA with Tukey's multiple comparisons test. (G-J) Representative whole-mount images and quantification of vascularized retinal area, radial outgrowth of P7 retinas from DMSO or SC79 treated *Klf9^R-loxp^* (n = 6 for DMSO, n = 8 for SC79) and *Klf9^iEC-Tg^* (n = 6 for DMSO, n = 7 for SC79) stained with IB4 (red, vasculature). *p < 0.05, **p < 0.01, ***p < 0.001, mean ± SEM, one-way ANOVA with Tukey's multiple comparisons test.

To evaluate the functional relevance of the Klf9/PI3K-Akt axis *in vivo*, we further assessed retinal vascular development in endothelial-specific Klf9 knockout (*Klf9^ΔEC^*) and overexpression (*Klf9^iEC-Tg^*) mice, with Akt modulation via MK2206 or SC79 administration (Fig. 10A, B). Western blot analysis demonstrated that the p-AKT/AKT ratio was increased in DMSO-treated *Klf9^ΔEC^* mice compared with *Klf9^fl/fl^* mice, and this effect was rescued by MK2206 treatment (Supplementary Fig. 5A, B). Notably, in *Klf9^ΔEC^* mice, the enhanced vascularization and proliferation phenotypes were suppressed by the Akt inhibitor MK2206 (Fig. 10C-F).

Conversely, Western blot analysis demonstrated that the p-AKT/AKT ratio was decreased in DMSO-treated *Klf9^iEC-Tg^* mice compared with *Klf9^R-loxp^* mice, and this effect was rescued by SC79 treatment (Supplementary Fig. 5C, D). Similarly, in *Klf9^iEC-Tg^* mice, which exhibited impaired retinal vascularization, SC79 treatment restored the vascularized area and vascular outgrowth on P7 (Fig. 10G-J). Collectively, these data substantiate that Klf9 regulates retinal angiogenesis and endothelial function *in vivo*, primarily through the modulation of the PI3K-Akt signaling pathway.

## Discussion

The formation of a hierarchically organized and functionally competent retinal vasculature requires not only the initiation of angiogenic growth but also its timely resolution 45-49. Our study identified Klf9 as a stage-specific transcriptional brake that governs the transition from active angiogenesis to vascular stabilization in the developing retina. By integrating endothelial-specific genetic models, human disease data, and multi-omics analyses, we demonstrated that angiogenic resolution is not a passive consequence of declining pro-angiogenic signals, but rather an actively enforced transcriptional and epigenetic program.

The central finding of this study is the temporal and endothelial-restricted upregulation of Klf9 during postnatal retinal vascular remodeling. Klf9 expression sharply increases during the postnatal period, corresponding to the transition from active sprouting angiogenesis to vascular remodeling and maturation, and then stabilizes once the vascular network is established. Functional manipulation of Klf9 confirmed its role as a molecular brake; endothelial-specific deletion accelerated early vascular expansion, increased tip cell formation and endothelial proliferation, and reduced vascular regression, whereas endothelial-specific overexpression delayed vascular growth and enhanced regression.

Importantly, these phenotypes were largely normalized by P21, as assessed by vascular density measurements, indicating that Klf9 does not abolish angiogenesis but rather fine-tunes its tempo during a defined developmental window. The normalization of vascular development by P21 suggests the involvement of compensatory mechanisms that restore vascular homeostasis. Potential mediators include VEGF-dependent feedback regulation, as VEGF levels dynamically adjust to vascular demand during development 50. Additionally, upregulation of compensatory KLF family members, particularly Klf2 and Klf4, which promote endothelial quiescence and vascular integrity 51, may contribute to this recovery. Recent studies have also highlighted the importance of endothelial-pericyte crosstalk via VEGFR2 signaling in promoting vascular stabilization 52. Metabolic reprogramming of the vascular niche, such as shifts between fatty acid oxidation and glycolysis, can influence angiogenesis and vascular regeneration 53. These compensatory pathways likely reflect the robust plasticity of developing retinal vasculature. Taken together, these findings support the concept that retinal angiogenesis is governed by an active developmental checkpoint, at which Klf9 enforces the transition from growth to stabilization.

The Krüppel-like factor family has emerged as a group of master regulators that integrate mechanical, metabolic, and inflammatory cues to maintain endothelial homeostasis 12, 54-57. Unlike Klf2, Klf4, and Klf15, Klf9 exhibits markedly higher expression in the whole retina 33, although its endothelial-specific expression pattern parallels that of Klf2 and Klf4, with a progressive increase during early development and a peak at P15 6, the functional role of Klf9 in the retinal vasculature seems to be distinct. Previous studies have shown that Klf2 promotes vascular stability via Erk1/2-Klf2-S1pr1 signaling 58; whereas Klf4 regulates sprouting angiogenesis through DLL4-Notch signaling 11; and Klf15 suppresses angiogenesis via VASN-mediated Notch1 activation 12. In contrast, Klf9 has been characterized as a tumor suppressor and a negative regulator of neural development, where it exerts potent control over cell cycle progression 22, 59-61. In line with these established roles, our findings suggest that Klf9 may function in retinal ECs as a negative regulator of angiogenic activity. Mechanistically, this effect appears to be mediated, at least in part, through modulation of the PI3K-AKT pathway, thereby helping coordinate the transition from active angiogenesis to vascular quiescence. Together, these observations support the notion that Klf9 as a distinct member of the KLF family that functions primarily as a temporal repressor during angiogenic resolution rather than as a shear-stress-responsive or inflammatory regulator.

Klf9 has context-dependent functions in diverse biological systems. In glioma and hepatocellular carcinoma, Klf9 serves as a tumor suppressor by repressing miR-21 or activating p53 to inhibit proliferation 22, 23. In vascular pathology, Klf9 plays a protective role; its downregulation in arteriovenous fistulas reduces lncDACH1 expression, thereby activating the HSP90/SRPK1/p-AKT signaling axis and promoting neointimal hyperplasia 62. These complicated roles—tumor-suppressive in cancer and protective in vascular injury—highlight Klf9's remarkable versatility depending on the cellular context, disease type, and microenvironmental cues. Such context-dependent discrepancies likely reflect differences in the vascular bed and the metabolic environment. Our findings from *Klf9^∆EC^* and *Klf9^iEC-Tg^* mice further demonstrate that, within the highly specialized retinal microvasculature, Klf9 serves as a dominant constraint on endothelial activation.

Mechanistically, our study revealed that Klf9 regulates retinal angiogenesis through chromatin-mediated repression of a pro-angiogenic transcriptional program encompassing PI3K-Akt signaling and focal adhesion-associated genes. While PI3K-Akt signaling has long been recognized as a central driver of endothelial cell proliferation, migration, and survival, its regulation has largely been viewed through the lens of receptor activation and kinase phosphorylation. Our integrated RNA-seq, ATAC-seq, and ChIP-qPCR analyses demonstrated that Klf9 operates at a higher regulatory tier by directly restricting chromatin accessibility to the promoters of key pathway components, including AKT1, PTK2, RAC1, and associated cytoskeletal and adhesion regulators.

Our data indicate that Klf9 regulates the AKT pathway in retinal endothelial cells selectively through Akt1 transcription. Specifically, Klf9 altered chromatin accessibility and reduced the expression of Akt1, while Akt2 and Akt3 levels were not significantly changed. This isoform-selective effect is pertinent because Akt1 is the predominant endothelial AKT isoform and has a well-established role in developmental angiogenesis 24, 40. Therefore, Klf9-mediated suppression of Akt1 provides a plausible molecular explanation for the suppressed angiogenic phenotype.

Furthermore, this mechanism clarifies how Klf9 integrates with known regulators of retinal angiogenesis to orchestrate vascular development. During active angiogenesis, cues such as VEGF strongly drive endothelial proliferation and sprouting via the PI3K-AKT axis 39. We propose that Klf9 acts as a crucial transcriptional brake within this network. As vessels mature and experience dynamic changes in the microenvironment, the upregulation of Klf9 limits Akt1 availability, thereby facilitating the transition from active vessel growth to quiescence. This function complements the roles of other key vascular regulators; for instance, while Klf2 promotes vascular stability primarily through S1pr1 signaling 56, 58 and Klf4 coordinates tip-stalk cell behavior via the DLL4-Notch pathway 11, Klf9 specifically modulates the amplitude of AKT-driven angiogenic signals. Together, these complementary mechanisms ensure a balanced progression from robust angiogenesis to a mature, stable vascular network.

The relevance of this regulatory axis extends from development to pathological angiogenesis. In ischemic retinal diseases, such as PDR and OIR, we observed a marked downregulation of Klf9 in endothelial cells, accompanied by excessive endothelial proliferation and the formation of immature, leaky neovascular tufts. Endothelial-specific loss of Klf9 exacerbates these pathological features, whereas its overexpression confers significant vascular protection. Notably, both *in vitro* and *in vivo* rescue experiments demonstrated that pharmacological modulation of Akt activity was sufficient to reverse the vascular phenotypes induced by Klf9 manipulation, supporting Akt as a critical downstream effector of Klf9 in retinal angiogenesis. These observations are directly related to retinal vascular diseases. Pathological neovascularization coexists with vascular defects and leakage in both diabetic retinopathy (DR) and retinopathy of prematurity (ROP). The downregulation of Klf9 in ischemic retinas suggests that the loss of this endothelial brake may contribute to the uncontrolled angiogenesis and persistent vascular immaturity characteristic of these diseases. In the absence of Klf9-mediated restraint, endothelial cells likely exhibit heightened sensitivity to pro-angiogenic stimuli, leading to excessive sprouting and persistent vascular instability, thereby allowing sustained activation of pro-angiogenic transcriptional programs, including the PI3K-Akt pathway identified as a direct target of Klf9. This loss exacerbates the formation of fragile, leaky neovessels and impairs vessel maturation. Thus, restoration of Klf9 function may represent a novel strategy for promoting vascular normalization and limiting disease progression.

These findings have significant therapeutic implications. Current anti-VEGF therapies primarily suppress angiogenic initiation, but do not directly address the failure of vascular maturation and stabilization, which characterizes pathological neovascularization. In contrast, targeting Klf9or its downstream chromatin-encoded regulatory program has the potential to restore the balance between endothelial growth and stabilization, thereby limiting aberrant angiogenesis while promoting vascular integrity. Such an approach may complement existing anti-VEGF strategies and help overcome their limitations in diseases characterized by persistent vascular leakage and immaturity.

The present study had several limitations that warrant consideration. Although this study focused on endothelial-intrinsic Klf9, our single-cell data revealed its expression in Müller glia and pericytes, suggesting its potential involvement in intercellular crosstalk within the neurovascular unit. Müller cells respond to metabolic stress by secreting angiogenic factors such as VEGF 63, 64, and given Klf9's role in regulating inflammation and cell death 65, glial Klf9 could modulate the secretome to indirectly influence vascular homeostasis. Similarly, pericytes are critical for vascular stability via direct EC communication 66, and Klf9 in pericytes may affect endothelial function by modulating pericyte recruitment or contractility. Future studies using cell-type-specific conditional knockouts are necessary to elucidate the non-endothelial contributions of Klf9. In addition, although our data established a critical role for Klf9 in the regulation of retinal angiogenesis, whether similar regulatory mechanisms operate in other vascular beds or disease contexts remains to be determined. Finally, although endothelial-specific modulation of Klf9 confers vascular protection in experimental models, the long-term consequences, safety, and therapeutic feasibility of targeting this pathway in patients require careful evaluation.

In summary, our study establishes Klf9 as a stage-specific transcriptional brake that coordinates angiogenic growth with vascular stabilization through repression of the PI3K-Akt signaling axis. These findings redefine angiogenic resolution as an actively enforced process, and identify a chromatin-encoded regulatory mechanism with broad implications for vascular development and disease. By elucidating how endothelial cells terminate angiogenic programs, this study provides a conceptual framework and a potential therapeutic entry point for pathological retinal neovascularization.

## Supplementary Material

Supplementary figures and table.
