# Supplementary material for: Endothelial Klf9 fine-tunes Akt signaling to act as a transcriptional brake restraining retinal angiogenesis
Source: Int J Biol Sci. 2026 May 11;22(10):5203–27. doi: 10.7150/ijbs.133293 (PMC13215252; doi:10.7150/ijbs.133293)
Supplement: Supplementary file 1 — Supplementary figures and table. [file ijbsv22p5203s1.pdf]

Supplementary figure 1

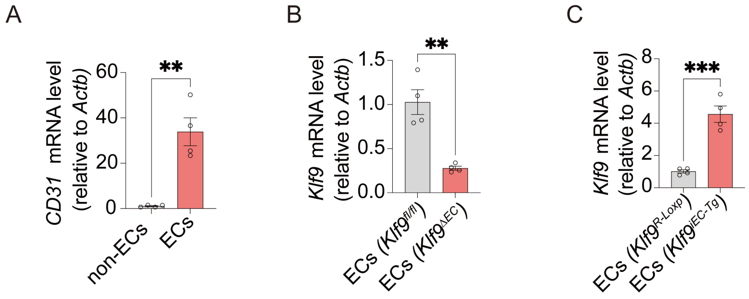

Supplementary Fig. 1 Establishment of endothelial-specific Klf9 deletion and overexpression in mice. (A) RT-qPCR confirmed the efficiency of magnetic bead sorting of CD31-positive cells from mouse retina (n = 4 per group). $**p < 0.01$, mean ± SEM, two-tailed unpaired Student's t test. (B) RT-qPCR confirmed the efficiency of endothelial-specific deletion of Klf9 from $Klf9^{\Delta EC}$ mouse ECs compared with $Klf9^{fl/fl}$ controls (n = 4 per group). $**p < 0.01$, mean ± SEM, two-tailed unpaired Student's t test. (C) RT-qPCR confirmed the efficiency of endothelial-specific overexpression of Klf9 from $Klf9^{iEC\text{-}Tg}$ mouse ECs compared with $Klf9^{R\text{-}loxp}$ controls (n = 4 per group). $***p < 0.001$, mean ± SEM, two-tailed unpaired Student's t test.

Supplementary figure 2

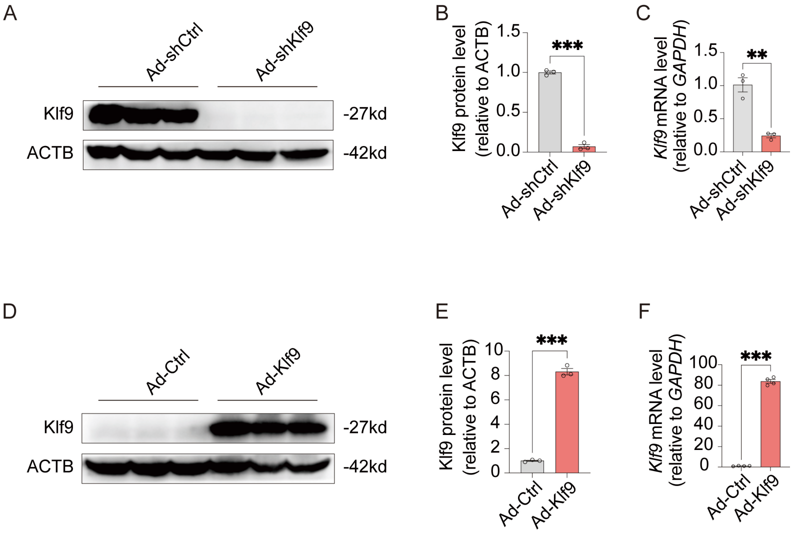

Supplementary Fig. 2 Establishment of Klf9 deletion and overexpression in HRMECs. (A, B) Quantification of Klf9 protein levels relative to GAPDH in the HRMECs transduced with Ad-shCtrl and Ad-shKlf9 (n = 3 biological replicates per group). ***$p < 0.001$, mean ± SEM, two-tailed unpaired Student's t test. (C) RT-qPCR confirmed the efficiency of endothelial-specific deletion of Klf9 in HRMECs (n = 3 biological replicates per group). **$p < 0.01$, mean ± SEM, two-tailed unpaired Student's t test. (D, E) Quantification of Klf9 protein levels relative to GAPDH in the HRMECs transduced with Ad-Ctrl and Ad-Klf9 (n = 3 biological replicates per group). ***$p < 0.001$, mean ± SEM, two-tailed unpaired Student's t test. (F) RT-qPCR confirmed the efficiency of endothelial-specific overexpression of Klf9 in HRMECs (n = 4 biological replicates per group). ***$p < 0.001$, mean ± SEM, two-tailed unpaired Student's t test.

Supplementary figure 3

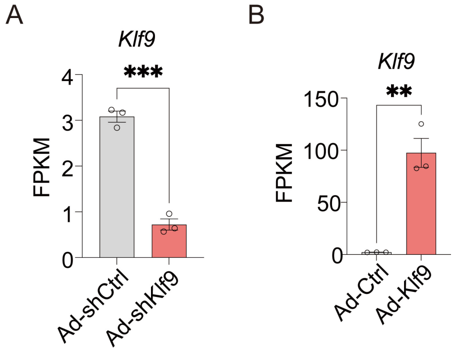

Supplementary Fig. 3 Validation of Klf9 knockdown and overexpression efficiency by RNA-seq. (A, B) FPKM values of Klf9 in HRMECs following knockdown or overexpression, as determined by RNA-seq (n = 3 biological replicates per group). **$p < 0.01$, ***$p < 0.001$, mean ± SEM, two-tailed unpaired Student's t test.

Supplementary figure 4

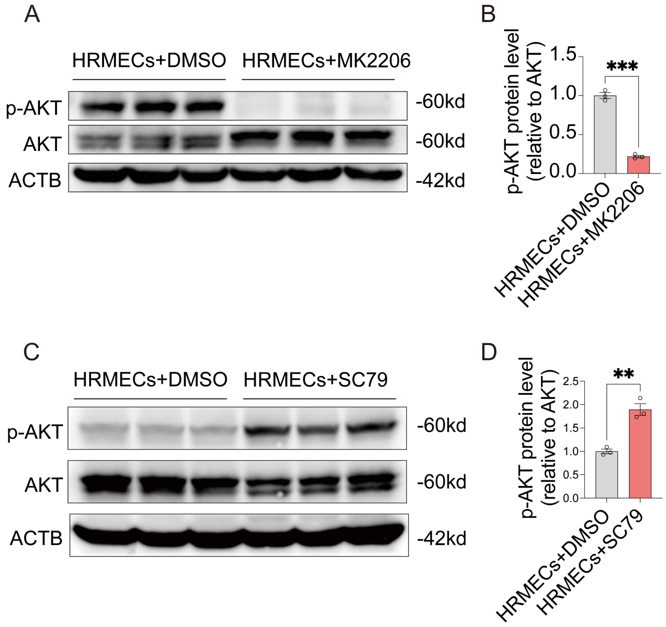

Supplementary Fig. 4 Establishment of Akt signaling activation and inhibition in HRMECs. (A, B) Quantification of p-AKT protein levels relative to AKT in HRMECs treated with DMSO and MK2206 (n = 3 biological replicates per group). ***$p < 0.001$, mean ± SEM, two-tailed unpaired Student's t test. (C, D) Quantification of p-AKT protein levels relative to AKT in HRMECs treated with DMSO and SC79 (n = 3 biological replicates per group). ***$p < 0.001$, mean ± SEM, two-tailed unpaired Student's t test.

Supplementary figure 5

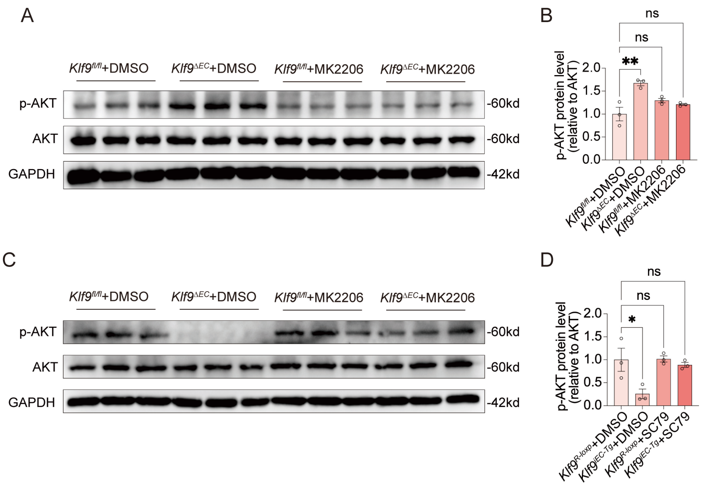

Supplementary Fig. 5 Modulation of Akt signaling by Klf9 deficiency or overexpression following MK2206 or SC79 treatment. (A, B) Quantification of p-AKT protein levels relative to AKT in *Klf9*$^{fl/fl}$+DMSO, *Klf9*$^{\Delta EC}$+DMSO, *Klf9*$^{fl/fl}$+MK2206 and *Klf9*$^{\Delta EC}$+MK2206 (n = 3 per group). $^{**}p < 0.01$, mean ± SEM, one-way ANOVA with Tukey's multiple comparisons test. (C, D) Quantification of p-AKT protein levels relative to AKT in *Klf9*$^{R\text{-}loxp}$+DMSO, *Klf9*$^{iEC\text{-}Tg}$+DMSO, *Klf9*$^{R\text{-}loxp}$+SC79 and *Klf9*$^{iEC\text{-}Tg}$+SC79 (n = 3 per group). $^{**}p < 0.01$, mean ± SEM, one-way ANOVA with Tukey's multiple comparisons test.

Supplementary Table 1. Primers for RT-qPCR and ChIP-qPCR.

Primers for RT-qPCR	Forward	Reverse
Mouse *Klf9*	CGAGCGGCTGCGACTACCTG	GGGCTGTGGGAAGGACTCGAC
Mouse *Actb*	GGCTGTATTCCCCTCCATCG	CCAGTTGGTAACAATGCCATGT
Mouse *Gapdh*	AGGTCGGTGTGAACGGATTTG	TGTAGACCATGTAGTTGAGGTCA
Mouse *CD31*	AGCCTAGTGTGGAAGCCAAC	AGCCTTCCGTTCTCTTGGTG
Human *Klf9*	GCCGCCTACATGGACTTCG	GGATGGGTCGGTACTTGTTCA
Human *GAPDH*	GGAGCGAGATCCCTCCAAAAT	GGCTGTTGTCATACTTCTCATGG
Human *AKT1*	AGCGACGTGGCTATTGTGAAG	GCCATCATTCTTGAGGAGGAAGT
Human *PTK2*	GCTTACCTTGACCCCAACTTG	ACGTTCCATACCAGTACCCAG
Human *RAC1*	ATGTCCGTGCAAAGTGGTATC	CTCGGATCGCTTCGTCAAACA
Human *VCL*	CTCGTCCGGGTTGGAAAAGAG	AGTAAGGGTCTGACTGAAGCAT
Human *MYL9*	TCTTCGCAATGTTTGACCAGT	GTTGAAAGCCTCCTTAAACTCCT
Human *RAPGEF1*	CTGAGGTGTCCGTAAAGATTCC	AGGGTAGCCCTCTGGTAGAAA
/	/	/
Primers for ChIP-qPCR	Forward	Reverse
Human *AKT1*	ATTCGTCCCTGACCTGTCTC	GGGCTGGGCCGCTAAC
Human *PTK2*	GACCCCAGGATGTCCGC	CTCGCGTAATTTGTCCTGTAGTG
Human *RAC1*	CTCGTGACCTCAGGTGATCC	CGCTCCCGGAGTTTCTCTG
Human *VCL*	GGGCAATCATACCTAGTTCCA	GCCCTCCTCAAAGACATTCTG
Human *MYL9*	CCCAGAGCGAACCCCCAC	CCTTATTTGGCCTCGACCCAC
Human *RAPGEF1*	CAGAGTCACCAGCTCCAGAC	ACGTCCTGGGGTGGAAAATC
